# Optimal Sampling Strategies for Detecting Zoonotic Disease Epidemics

**DOI:** 10.1371/journal.pcbi.1003668

**Published:** 2014-06-26

**Authors:** Jake M. Ferguson, Jessica B. Langebrake, Vincent L. Cannataro, Andres J. Garcia, Elizabeth A. Hamman, Maia Martcheva, Craig W. Osenberg

**Affiliations:** 1 Department of Biology, University of Florida, Gainesville, Florida, United States of America; 2 Department of Mathematics, University of Florida, Gainesville, Florida, United States of America; 3 Emerging Pathogens Institute, University of Florida, Gainesville, Florida, United States of America; 4 Department of Geography, University of Florida, Gainesville, Florida, United States of America; University of New South Wales, Australia

## Abstract

The early detection of disease epidemics reduces the chance of successful introductions into new locales, minimizes the number of infections, and reduces the financial impact. We develop a framework to determine the optimal sampling strategy for disease detection in zoonotic host-vector epidemiological systems when a disease goes from below detectable levels to an epidemic. We find that if the time of disease introduction is known then the optimal sampling strategy can switch abruptly between sampling only from the vector population to sampling only from the host population. We also construct time-independent optimal sampling strategies when conducting periodic sampling that can involve sampling both the host and the vector populations simultaneously. Both time-dependent and -independent solutions can be useful for sampling design, depending on whether the time of introduction of the disease is known or not. We illustrate the approach with West Nile virus, a globally-spreading zoonotic arbovirus. Though our analytical results are based on a linearization of the dynamical systems, the sampling rules appear robust over a wide range of parameter space when compared to nonlinear simulation models. Our results suggest some simple rules that can be used by practitioners when developing surveillance programs. These rules require knowledge of transition rates between epidemiological compartments, which population was initially infected, and of the cost per sample for serological tests.

## Author Summary

Outbreaks of zoonoses can have large costs to society through public health and agricultural impacts. Because many zoonoses co-occur in multiple animal populations simultaneously, detection of zoonotic outbreaks can be especially difficult. We evaluated how to design sampling strategies for the early detection of disease outbreaks of vector-borne diseases. We built a framework to integrate epidemiological dynamical models with a sampling process that accounts for budgetary constraints, such as those faced by many management agencies. We illustrate our approach using West Nile virus, a globally-spreading zoonotic arbovirus that has significantly affected North American bird populations. Our results suggest that simple formulas can often make robust predictions about the proper sampling procedure, though we also illustrate how computational methods can be used to extend our framework to more realistic modeling scenarios when these simple predictions break down.

This is a *PLOS Computational Biology* Methods article.

## Introduction

The effectiveness of disease control measures often depend on when outbreaks are first discovered. Early detection can significantly reduce the costs associated with disease eradication, human illnesses, and devastation of livestock or crops. For example, the 2001 epidemic of foot and mouth in Great Britain was reported only 2 weeks after the epidemic began [1] yet had an estimated financial impact of $11.9–$18.4 billion dollars [2]. A hypothetical foot and mouth epidemic in California not detected for 2 weeks could have a financial impact of over $15 billion dollars, and an epidemic not detected for 3 weeks could have an impact of up to $69 billion dollars [3]. Although many studies have examined alternative control strategies and the impact of detection time on control [1], [2], [4], the complementary question of how to achieve early detection has been relatively neglected by theory. Greater attention to the design of disease surveillance methods may facilitate earlier detection and reduce the economic impacts of disease epidemics.

Passive surveillance methods are the voluntary reporting of cases by primary care providers and citizens to public health officials [5]. Recent work on passive surveillance methods for human infectious diseases has progressed rapidly and includes developing methods to optimize the placement [6], [7] and performance [8] of surveillance sites. Integrating these physical surveillance systems with internet search data has led to improvements in the performance of traditional physical reporting systems [7], [9]. Active surveillance methods of zoonotic diseases are the periodic sampling by health authorities [5]. For vector-borne diseases active surveillance may include the use of sentinel animals and the longitudinal sampling of vector populations [10]. Active surveillance may often perform better for targeted objectives than passive methods [5], and recent work has begun to link active zoonotic surveillance data to epidemiological models. For example, Gerardo-Giorda *et al.* [11] combined surveillance data and epidemiological models to identify counties that were most important for surveillance efforts of rabies in New York State. It is likely that analytical approaches will prove useful in making active zoonotic surveillance methods more cost effective, an important consideration for surveillance organizations with limited resources [12].

Past analytical work on active disease detection examined how sampling for infected individuals in a susceptible population affects the time at which an epidemic is detected [13], [14] and the subsequent incidence of a disease at the time of discovery [15]. These studies have examined the dynamics of diseases that are directly transmitted and thus lack a disease vector. As a result, we still have little knowledge to guide early detection theory for zoonotic diseases (e.g., Lyme disease, malaria, Rift Valley fever virus, West Nile virus, dengue fever), where sampling could occur in vector populations or host populations. Here, we studied the optimal sampling design for early disease detection using formulations of a disease with one host population and one vector population. We combined models of host-vector dynamics with a periodic sampling procedure in which sample size is constrained by economic limitations. We used a susceptible-infected (SI) model to examine how to allocate sampling effort between the vector and host populations, and we used a susceptible-infected-recovered (SIR) model to look at allocating sampling effort between the vector population, infected hosts, and recovered hosts.

The CDC guidelines for evaluating public health surveillance of human based diseases [16] are standards that have been used in many assessments of zoonotic surveillance systems, although differences may exist between human and zoonotic surveillance goals [17]. A recent survey on the assessment of surveillance systems found that a number of different metrics have been used to determine zoonotic surveillance performance; two of the most frequently mentioned criteria are the sensitivity of surveillance (the ability to detect outbreaks or infection rates) and the time to outbreak detection from initial exposure [17]. Here we assume the goal of surveillance is to detect the outbreak as early as possible to minimize financial damages or spillover human infections, a common goal for zoonoses [12]. Our results provide some basic rules of thumb for practitioners designing active surveillance protocols for vector-borne diseases.

## Models

We modeled the early dynamics of a disease with a single vector species and a single, non-human host species. Here we define early-time approximations to systems where vectors follow SI dynamics and hosts follow either SI- or SIR- dynamics. We then define a sampling model that can be applied to these dynamical systems. The sampling of human hosts often has additional considerations not accounted for in this sampling framework. We therefore address specific issues about human populations in the discussion.

### Vector-host SI model

We made assumptions common to other SI models of vector-borne diseases: vectors and hosts can be in either a susceptible or an infected state at time 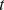, the disease epidemic (the dynamics of interest) occur on a relatively short time-scale and thus infected individuals cannot recover nor do individuals give birth or die over the course of the epidemic, and infection spreads only through interspecific interactions [18], [19]. Subscripts are used to denote population-level parameters: e.g., 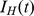 and 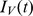 denote the number of infected hosts (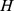) and vectors (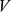) at time 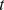, respectively (Table 1). These assumptions give the following system of equations for the dynamics of infection of the SI model:
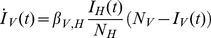
(1a)

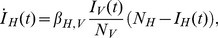
(1b)where 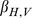 and 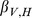 correspond to the disease transmission rates from vectors to hosts and hosts to vectors, respectively. 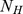 and 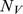 correspond to the total host and vector population sizes, respectively. Throughout this work we assume that population sizes are constant over the course of the epidemic and that individuals are in the population only if they can potentially contract the disease. This implies that individuals that are epidemiologically isolated are not a part of the population. Note that the dynamics of susceptible host (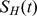) and vector (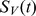) populations are completely determined by system (1) because 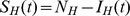 and 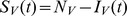.

**Table 1:** Descriptions of parameters and sources of parameter estimates.

Parameter	Description	Value	Source
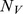	Abundance of vectors.		
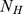	Abundance of hosts.		
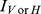	Number of infected individuals in the vector or host populations.		
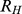	Number of recovered individuals in the host population.		
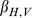	Daily transmission rate from vectors to hosts.	0.0792	[26]
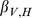	Daily transmission rate from hosts to vectors.	0.0144	[26]
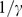	Number of days until infected host recovers.	varied	
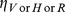	The economic efficiency of the vector, infected host, or recovered host populations.		
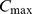	Resources allocated to obtaining and running samples in a fixed period of time.	88	in text
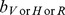	Cost of running a sample from the vector population, infected host population, or recovered host population.	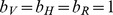	in text
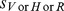	Sample size of the vector population, infected host population, or recovered host population.		
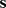	Optimal sample design for a epidemiological system that is sampled with economic constraints.		
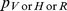	Pool sizes of the vector population, infected host population, or recovered host population.	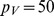 , 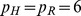	in text
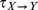	Critical time in a linearized system. At this time the optimal sampling design changes from stratum 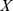 to stratum 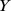 .		

Because we are interested in detecting a disease as early as possible, we focus on the dynamics of the system immediately after disease introduction. Therefore we linearized system (1) about the disease-free state 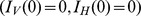 and obtained:
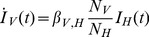
(2a)

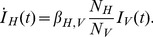
(2b)We focus our subsequent analyses on the specific scenario of an epidemic started by an infected host with the initial conditions 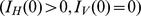 though analyses of alternative initial conditions 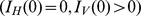 are presented in Text S3 and S4. Because of the symmetric nature of system (2), this analysis yields similar results. With the assumptions listed above, the solution to system (2) is:
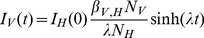
(3a)

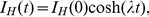
(3b)where 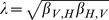.

### SIR host- SI vector model

For the SIR model we assume that recovered hosts obtain immunity over the timescale of the epidemic. As in the SI model the disease cannot spread through direct contacts within host and vector populations, transmission is frequency-dependent, and individuals are not born and do not die over the course of the epidemic. The full model for a single host population and single vector population is given by
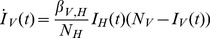
(4a)

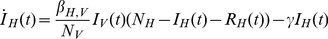
(4b)

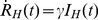
(4c)where 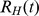 designates recovered individuals and 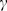 is the recovery rate of infected individuals. Note that the dynamics of susceptible host (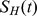) and vector (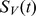) classes are completely determined by system (4) because 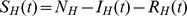 and 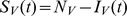. The corresponding linearized model evaluated at 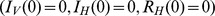 is
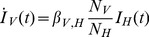
(5a)

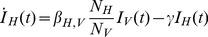
(5b)

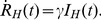
(5c)For an epidemic begun by an infected host 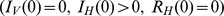, the solution of (5) is:
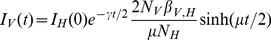
(6a)

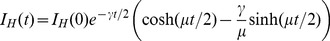
(6b)

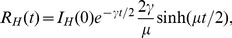
(6c)where 
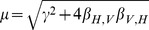
. Solutions for the alternative initial conditions 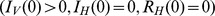 for system (5) are given in Text S4. This analysis is slightly more complicated than that presented in the main text, but the core ideas remain the same.

### Sampling model

Consider sampling at time 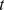 from a population with potentially infected hosts and vectors. Let 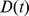 denote the set of events such that the disease is detected from a sample of size 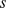. If the total population abundance is much greater than the sample size then 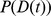, the probability of detecting the disease, can be modeled as a binomial random variable. When the sample size is comparable to the population abundance, the hypergeometric distribution is a suitable sampling model. We do not consider the hypergeometric model here as the binomial distribution provides a reasonable approximation for realistic sample sizes. The proportion of infecteds at time 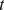 is given by 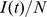. In a sample of size 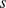, 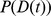 is the complement of not detecting any infected individuals,
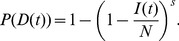
(7)When the disease is rare (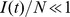) equation (7) is well approximated as
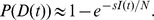
If there are two sampling strata (e.g., a host and vector, although the approach works as well for two host species or two strata of hosts in a single species), we need the probability of detecting the disease in either of those strata, 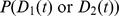. For two strata this quantity is given by
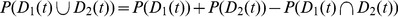
(8)

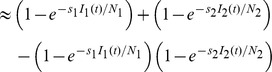


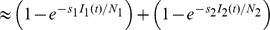


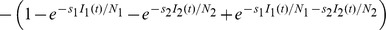


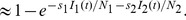
(9)A more general form when there are 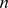 sampled strata is given by
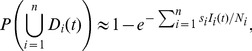
(10)as shown in Text S1.

We also consider the probability of detecting the disease for the first time in the 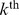 sample when sampling occurs regularly at discrete time intervals. A model of detecting the disease in the current sampling period, but not before, is a geometric distribution with time-dependent detection probabilities:
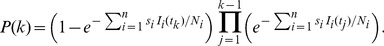
(11)Here the number of infecteds in stratum 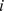 in the 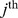 sampling period is given by 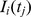. Sampling strata are defined by both the animal population being sampled and the type of test that is run. For example, immunological tests on bird populations for West Nile virus can test whether individuals are currently infected or have been previously infected by the type of antibody present in the sample. Antibody-specific tests therefore distinguish between infected birds and recovered birds. The first term on the right-hand side of expression (11) represents the probability of detecting the disease in time period 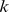. The remaining 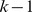 terms (given in capital Pi notation, 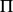) represent the probability of not detecting the disease in sampling period 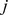, where 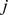 runs from 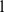 to 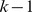. The product of these 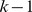 terms gives the probability of not detecting the disease in any of the 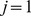 to 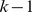 sampling periods. We minimized the time until detection of the epidemic using the geometric probability distribution defined in equation (11) and by using the expected time to detection, given by
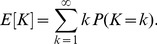
(12)This expected value is an infinite series that converges to an unknown quantity, therefore we numerically approximated 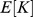.

We have so far considered the possibility of sampling and testing infected and recovered individuals in populations. However, a common practice in zoonotic surveillance is to combine samples from multiple individuals in the stratum of interest in order to save money (e.g., [20]). Though this pooled sampling does not identify which individual tested positive for the virus, the goal of surveillance is often to identify the presence of the virus instead of a specific infected individual. Pooling sizes must be constrained to prevent the possibility of a positive individual sample being diluted below detectable levels, often determined using experimental dilutions in the laboratory (e.g., [21]). To incorporate pooled sampling into our sampling model, we rescaled the probability of a positive detection in a single sample by the number of individuals in a pool, 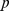: the probability of detection in a single *pooled* sample of strata 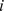 at time 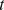 can then be approximated using a linearized binomial expansion:
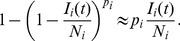
(13)Approximation (13) works well when 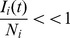. Our simulations indicate that a 10% approximation error occurs when 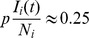, suggesting the approximation is robust for the purpose of early detection. Pooled sampling modifies (10) and (11) to
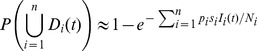
(14a)

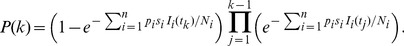
(14b)Our goal is to determine the resource allocation that will allow us to detect a disease as early as possible. We therefore introduce economic constraints on this sampling process in the next section.

### Economic constraints and optimization

Agencies are often faced with monitoring endemic and emerging diseases with finite resources. This necessitates allocating those resources in the most efficient way possible. We applied a cost function to describe these constraints: we let 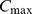 be the budget for a set of samples taken periodically and 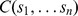 be the cost of sampling 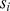 individuals from stratum 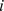, 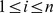. If we assume that we spend our entire budget then 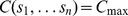. For example, a linear cost function for a vector stratum and a host stratum can be written as
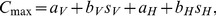
(15)where 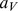 and 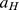 are the overhead costs (operating costs) associated with sampling vectors and hosts respectively, while 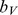 and 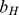 are the corresponding costs per sample.

We used the Karush-Kuhn-Tucker (KKT) conditions [22] to find the sampling strategy 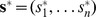 that maximizes the probability of detection (given by equation (14a)) or minimizes the time to disease detection (given by equation (14b)). The KKT approach allows the minimization of a function subject to inequality constraints, e.g., constraining the sample sizes to be nonnegative. Further details on this method, as well as some general results for cases with linear objective functions, are provided in Text S2 and S3.

## Results

Here we apply the sampling framework defined in the Methods to hypothetical epidemics of West Nile virus. We provide some analytical results and examine via computer simulation how well our early-time approximations match the corresponding full models.

### SI dynamics

#### A general solution to the host-vector SI model

We now determine the sampling scheme that maximizes the probability of disease detection under the assumptions of the full SI model for one host and one vector species, given by system (1). To find potential optimal sampling schemes in the SI host-vector model, 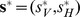, we maximize the probability that the disease will be detected in a single sampling trial at a fixed time 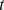, assuming that the states 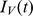 and 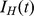 are known. When applying the KKT conditions to equations (10) or (11), we see that the optimal sampling scheme depends on a quantity that we call the economic efficiency of a stratum. The economic efficiency of stratum 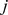 is
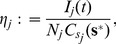
(16)where 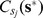 is the partial derivative of the cost function with respect to the sample size of the 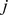
th stratum evaluated at the optimal strategy 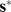. The economic efficiency, 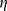, gives the marginal return on a dollar investment: i.e., it gives the expected number of new positive detections for a small change in added investment. This quantity, derived in Text S3, is useful for testing the conditions needed to determine the optimal sampling design.

With reference to Text S3, and making no assumptions about the form of the cost function 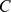, we see that there are five possible types of optimal sampling scheme. First, it is possible that there is no nontrivial optimal sampling sampling scheme. This would be the case if the total overhead cost was as large or larger than the budget (i.e. 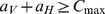). Next, if the disease is not present, all economically feasible sampling schemes give the same (zero) probability of detection. Lastly, if neither of these cases holds, then there exists some nontrivial optimal sampling scheme 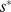. The KKT conditions give candidates for 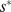. All candidates must then be evaluated to determine the true optimal sampling scheme 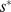. Candidates for the nontrivial optimal sampling scheme at a fixed time 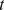 are determined by comparing 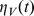 and 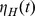:

First, if there exists some 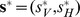 such that 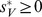, 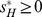 and if
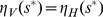
(17)then 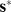 is a candidate for the optimal sampling scheme. This sampling scheme would involve sampling from both the vector and the host populations. Second, if there exists some 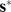 such that 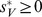, 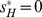 and if
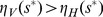
(18)then 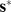 is a candidate for the optimal sampling scheme. This sampling scheme would entail sampling from only the vector population. Third, if there exists some 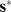 such that 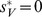, 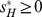 and if
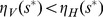
(19)then 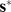 is a candidate for the optimal sampling scheme. This sampling scheme would entail sampling from only the host population. Note that for a nonlinear cost function, it is possible that more than one of (17)–(19) may hold at the same time for different sampling strategies. Once all candidates for 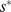 have been found, the probability of detection (given in (10)) must be evaluated for each candidate and maximized.

In the case of a linear cost function, that is, when 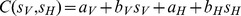, the partial derivatives 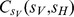 and 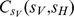 are constants. Thus, the expressions for 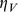 and 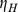 are independent of 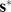:
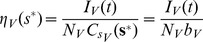


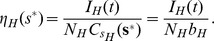
Now, since at any time 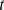 only one of the relations (17)–(19) can hold, there is only one candidate for the nontrivial sampling scheme 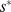 and we have that the relative magnitudes of 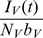 and 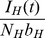, or equivalently 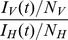 and 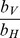, determine the nontrivial optimal sampling scheme 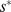. Thus, we deduce the shape of the curve 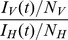 with respect to time 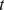. A complete treatment of this problem is given in Text S3. Here, we give an outline of the solution, though the rest of this section can be skipped by readers uninterested in this level of mathematical detail.

To simplify our notation in the following analysis, we define
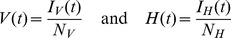
(20)to be the proportion of the vector (host) population that is infected. We are then concerned with the curve 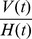. Noting that whenever 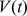 is positive 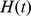 is strictly increasing with respect to time 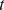, we reparameterize 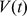 as a function of 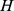 to obtain 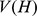. We now see that our original problem is equivalent to characterizing the curve 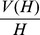 with respect to 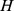. Thus we can rewrite the conditions outlined in equations (17)–(19) as:
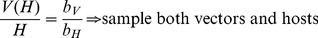
(21)

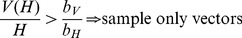
(22)

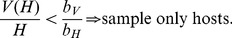
(23)


**Figure 1:**
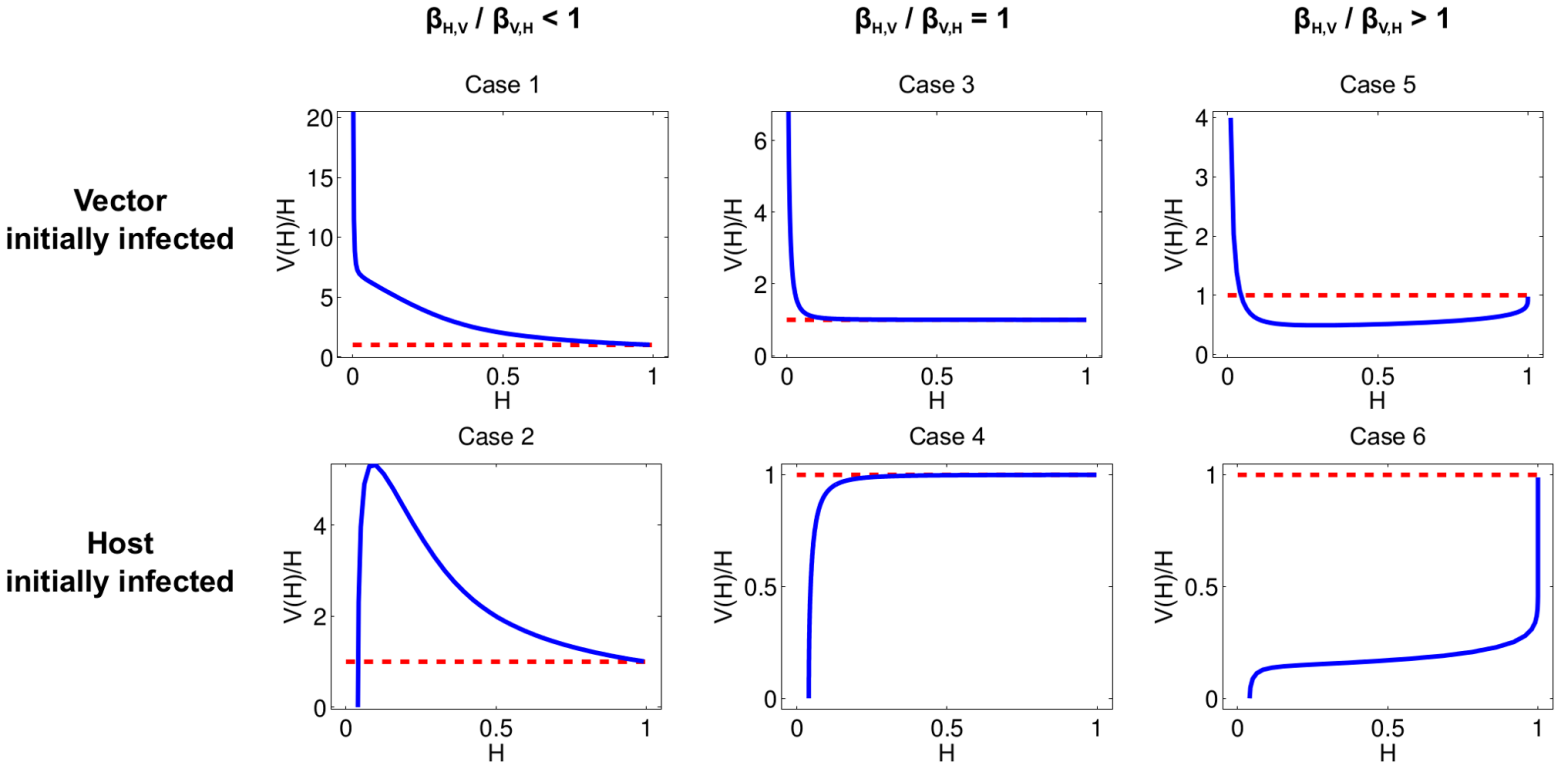
The relative magnitudes of 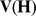 and 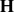 under six cases that differ in initial condition and relative transmission rates. The position of 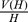 (above or below) relative to the red dashed line (of height one) remains the same for any choice of 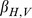 and 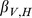 within each case shown. The parameters used are as follows. Case 1: 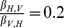, 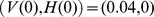; Case 2: 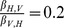, 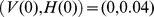; Case 3: 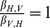, 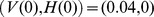; Case 4: 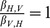, 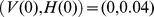; Case 5: 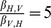, 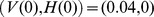; Case 6: 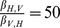, 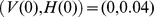. Note that smaller initial conditions do not change the qualitative behavior shown. Instead, as 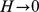, the blue curve would approach the origin in Cases 2, 4, and 6, and the blue curve would approach positive infinity in Cases 1, 3, and 5.

The curve 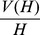 can be completely characterized by the ratio 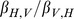 and the initial conditions (Text S3). The three relative states of 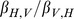 (i.e., 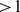, 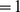, or 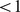) and the two initial conditions (i.e., the disease starts in the vector vs. the host) define six qualitatively different curves for 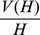 (Figure 1). It is important to note that for fixed sampling costs 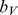 and 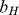, the optimal sampling scheme depends only on the proportion of a population that is infected (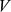 and 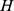). Thus the number of infecteds (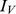 and 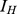) and the time since introduction affect the optimal sampling scheme only via their influence on the proportion of a population that is infected.

We can use Figure 1 to elucidate the optimal sampling scheme at a given time. If the curve 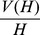 lies above 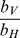 (i.e. satisfies (22)), then the optimal scheme is to sample only vectors. Conversely, if the curve 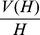 lies below 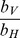 (i.e. satisfies (23)), then the optimal scheme is to sample only hosts. In all six cases, the best sampling scheme at very early times (corresponding to a small proportion of the host population being infected, i.e. small 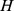) is to sample the population in which the epidemic originated; however, as the epidemic progresses, it is possible to have a switch to sampling the population that was not initially infected.

Lastly, we consider the effect of sampling error on our determination of optimal sampling scheme. Suppose there is a small error 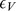 (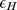) in the detected proportion of infected vectors (hosts). Then by equations (21)–(23), the optimal sampling scheme is determined by comparing the relative magnitudes of 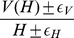 and 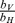. Early in the progression of the epidemic, both 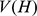 and 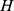 may be very small, even on the same order of magnitude as the errors 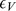 and 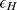. In this case, sampling error can easily alter the determined optimal sampling scheme. As the disease progresses, 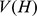 and 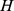 become larger and the perturbation by 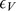 (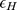) becomes less significant. In the following example, we revert to our original notation, given in (20).

#### A general SI model of West Nile virus

We illustrate an application of sampling optimization by applying our approach to West Nile virus, a mosquito-borne pathogen introduced to the United States in 1999 [23]. Since introduction the disease has resulted in numerous deaths in humans [24] and large-scale declines in bird abundances [25]. Testing for West Nile virus has often relied on counts of dead birds. However, as pointed out by Hochachka et al. [10], this may only be useful for indicating the later stages of severe epidemics and thus fails to lead to effective containment of the epidemic. Here, we examine an alternative strategy wherein both mosquito traps and sentinel chickens, groups of chickens placed in cages and distributed throughout an area, are used to detect a disease epidemic. In the following analyses we investigate sampling between vector and bird host populations but neglect spillover hosts such as humans and horses.

Epidemiological parameter values are taken from the literature [26] or determined here; a summary is provided in Table 1. We assumed that the overhead costs were zero (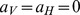) and that the cost of running a test is the same for a sample of mosquitoes or birds, which we set to the arbitrary value of 1 (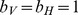). Mosquito samples are usually pooled into batches of around 50 individuals [20], while blood samples are pooled from birds in a single sentinel chicken cage, usually with 6 birds. Therefore we let 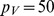 and 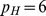. In the following analyses we assumed that the disease is introduced by the host, rather then the vector. This is likely a more common method of introduction for West Nile virus as birds typically move over much greater distances than mosquitoes (e.g., [27]).

We let 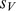 be the number of pooled vector samples tested and 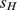 be the number of pooled host samples tested so that 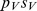 and 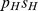 are the total number of individuals tested in the vector and host populations. Plugging the values defined above into (15) gives 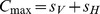, while the economic efficiencies for the vector and host populations are given by
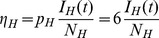
and
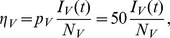
respectively. The slight modification of the economic efficiencies given in (16) includes the effect that pooling has on increasing the economic efficiency of sampling. We assumed a weekly budget commensurate with a county-level mosquito control agency. For example, the agency in Hillsborough County, Florida maintains 13 sentinel chicken flocks and 75 mosquito light traps that are typically checked weekly during peak season. Thus, we assumed that the weekly budget is 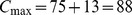.

We now use the analysis of the previous section to obtain some qualitative results regarding the aforementioned disease detection scenario. Recall that 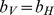, but that different numbers of mosquitoes (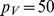) and chickens (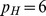) are pooled. As a result, the conditions given in (21)–(23) become:
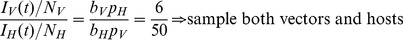


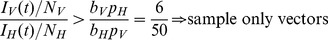


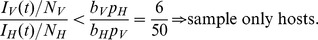
Note that from the parameter values given in Table 1 we have
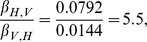
which is larger than one. Thus, (by Table 3 in Text S3) if a vector is initially infected, the curve 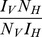 is qualitatively similar to that shown in Case 5 of Figure 1. If a host is initially infected, the curve 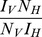 is qualitatively similar to that shown in Case 6 of Figure 1.

Suppose that a host is initially infected. (Figure 1, Case 6) Then 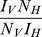 is close to zero at early times, so the above conditions imply that the optimal sampling strategy is to sample only hosts. As the infected vector population grows and becomes larger than 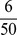, there is a switch from sampling only the host population to sampling only the vector population. Since this switch depends on the ratio of the proportions of the populations that are infected, the switch will occur at different times for different total population sizes, assuming a constant number of initially infected individuals and constant transmission rates. Note that if it is more expensive to sample the host population than the vector population (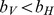) then the time to switch from sampling only hosts to sampling only vectors is earlier. Conversely, if it is more expensive to sample vectors (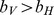) then the switch time becomes later.

It is easy to show that for the parameter values given in Table 1 if a vector is initially infected, then 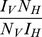 is always greater than 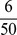 and the optimal sampling strategy is to expend the entire budget sampling vectors. If it becomes more expensive to sample hosts (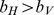), then the optimal sampling scheme does not change. Conversely, if it becomes sufficiently less expensive to sample hosts (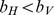) then there is a switch from sampling only vectors to sampling only hosts. As 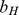 becomes smaller, the switch time becomes earlier. In the following section we show how to solve for the switch times using an approximation to this model.

#### Optimal sampling of the linearized SI model

Here we consider specific solutions of the linearized SI system, given by system (2), under the linear cost function described in the previous section: 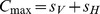. This simplified system can provide practitioners with some insight on sampling design when not much detailed information is known about the functional form of disease dynamics. When there is doubt about the validity of these approximations, the full solutions developed in the previous section can be used to determine the range over which linear approximations will be useful. Here and through the rest of the manuscript we only consider the introduction of disease by the more migratory host population, though calculations are provided for alternative initial conditions in Text S4.

Applying the KKT conditions (Text S2) to the linear solutions (3) shows it is optimal to place all of the sampling effort into either the host or vector populations when cost functions are linear. As above, we determined the stratum to sample by calculating and comparing the economic efficiencies of each stratum. The economic efficiencies with pooled sampling for this system are given by 
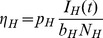
 and 
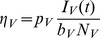
. With this model it is possible for the sampled population to switch at some critical time as discussed in the previous section, denoted 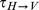, when the most economically efficient stratum changes from host to vector. If such a switch occurs, the critical time occurs when
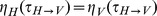


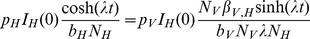


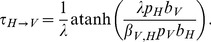
(24)Substituting 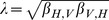 in the above equation, and since 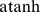 is defined only when its argument is less than one in absolute value, we see that 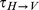 exists only if
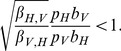
(25)Additionally, since 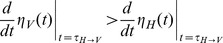 whenever 
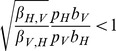
, vectors are the most economically efficient stratum at times later than 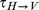. The critical time for the alternative case, 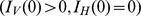, is given in Text S4.

#### The linearized SI sampling model applied to West Nile virus

We now revisit the example of West Nile virus using the linearized approximations derived in the previous section. First, the presence of a critical-time in the binomial sampling model was demonstrated because 
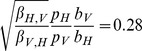
 (parameter values given in Table 1), which satisfied condition (25). Using (24), the critical time to switch from sampling hosts to vectors was 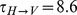 days. This is a short timescale when the sampling period is weekly; therefore, if the population is large then it is unlikely that the virus will be detected in the first week after initial infection and a reasonable time-independent strategy is to place most or all of the sampling effort into the vector population.

We also explored optimal sampling in the full SI model through simulation. This allowed us to determine how well our decisions based on binomial sampling with linearized dynamics approximated the full SI model with geometric sampling. We used the same parameter values as above but we varied both population abundances independently over several orders of magnitude. 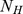 was varied from 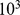 to 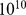 individuals and 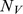 from 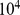 to 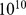 individuals. For each combination of population abundances considered, we simulated the dynamics of an SI epidemic using numerical solutions to the nonlinear system (1). We then calculated the expected time to disease discovery using (12) and tested each potential sampling strategy (the host sample size, 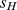, ranged from integers 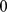 to 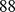 and the vector sample size was 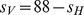). We found that the optimal sampling strategy, 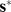, for all population abundances considered was dependent on 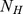 and independent of 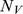. When 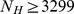 the optimal solution was 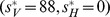, when 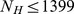 the optimal solution was 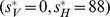, while within this relatively narrow range of abundances we found that a linear relationship described a mixed ideal strategy, where 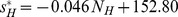 and 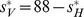. Slope and intercept coefficients were calculated from simulation output. This suggests that when the sample size, 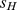, is less than 16% of the total population size, 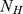, then the linearized system provides a reasonable approximation.

**Figure 2:**
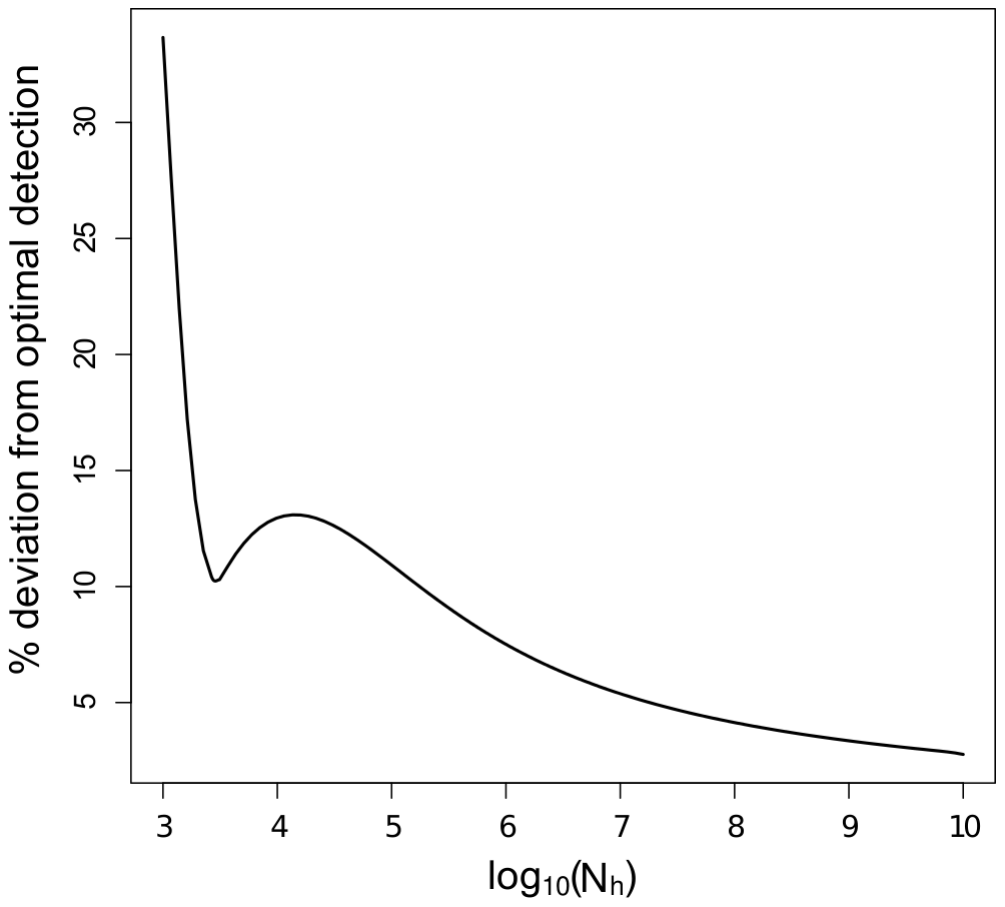
Percent difference in the expected detection time between the optimal, 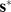, and suboptimal, 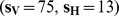, sampling schemes as a function of the host population abundance, 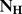. The vector sample size is denoted as 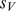, and the host sample size is denoted by 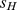.

Finally, we looked at the error due to suboptimal sampling by calculating the difference in time to detection of the epidemic by comparing the applied sampling design consistent with the parameters we inferred from the Hillsborough County mosquito control agency with 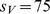, 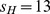 to the optimal sampling design 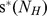. Proportional error levels were high when the vector population abundances were low, with an error of approximately 30% when 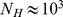, but this value quickly decreased (Figure 2). The absolute error in expected detection timing between the optimal and suboptimal sampling designs was about a week for all population abundances considered.

### SIR dynamics

#### A solution to the linearized SIR host- SI vector dynamics

In the case where the host population follows the SIR dynamics defined in (5) we must consider sampling between infected vectors (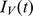), infected hosts (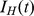), and recovered hosts (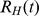). As a host progresses through the infected class and into recovery the relative quantities of virus and antibodies within the host change. Typically, different testing procedures are used to detect infected individuals and recovered individuals [28], [29]. Therefore, a framework to determine whether to sample for infected or recovered individuals can save resources and potentially lead to faster detection times. Here, using the expression for 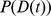 (10), the linearized solutions (6), and assuming a linear cost function (15), we solve for critical times at which the optimal sampling strategy changes.

We consider four critical times: 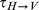, 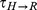, 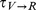, and 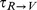. The first, 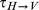, is the time at which the optimal sampling strategy switches from sampling only infected hosts to sampling only the vector population. The time at which the optimal sampling strategy switches from sampling infected hosts to sampling recovered hosts is 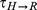. The times 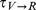 and 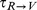 give the critical switches from sampling only the vector population to recovered hosts or vice-versa. The other potential critical times 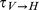 and 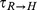 are not considered here because they do not exist at early times for for these initial conditions 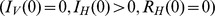, but expressions are given for alternative initial conditions in Text S4.

The first critical time, 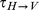, is given by the equivalence of the economic efficiencies for the infected vectors and hosts, 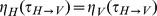. Similar to the process in the SI model we plug in 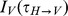 and 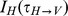 from (6) into the economic efficiencies and solve for 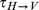. This gives
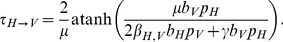
(26)Note that since the argument of 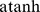 must be less than one in absolute value, 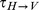 exists only if 
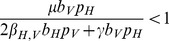
. Additionally, since 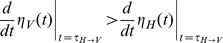 whenever 
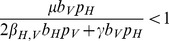
, vectors are the most economically efficient class at times later than 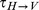. This formula is analogous to the SIS case given in equation (24), with some slight modifications due to the additional recovery state. As the recovery rate 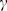 goes to 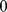, the above expression for 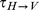 approaches the SIS formula given in (24).

The second case, 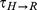, occurs when the economic efficiencies of infected and recovered hosts are equal, 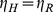. We first need to define 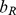 and 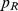, the cost per sample and pooling sizes of the recovered vector stratum. Plugging in solutions from (6) gives,
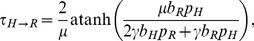
(27)where 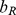 is the cost per sample for recovered hosts and 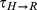 exists only if 
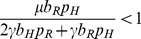
. Additionally, since 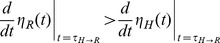 whenever 
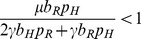
, recovered hosts are the most economically efficient class at times later than 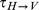.

The third and fourth cases are the switch from infected vectors to recovered hosts and vice versa. As before we set equal the economic efficiencies, 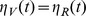, with appropriate substitutions for 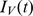 and 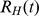. With the linearized solutions (6), the ratio of the economic efficiencies is a constant,
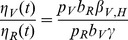
(28)


This means that 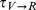 and 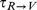 do not exist. Instead, the economic efficiencies determine which stratum is sampled by evaluating whether (28) is greater than or less than 1. If the ratio is greater than 1 it will be optimal to sample from the vector stratum; when less than 1 it is optimal to sample from the recovered host stratum. Critical times for an alternative initial conditions (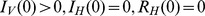) of the linearized SIR model with binomial sampling are provided in Text S4.

#### An SIR model of West Nile virus

We first applied the critical time expressions derived in the previous section using the linear approximation (6) and binomial sampling model (14a) to determine the optimal sampling design, 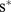. We then used simulations to determine if these values were consistent with the nonlinear SIR (4) and geometric sampling model (14b). We explore the optimal sampling design as a function of the unknown recovery time of the bird populations, 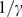.

**Figure 3:**
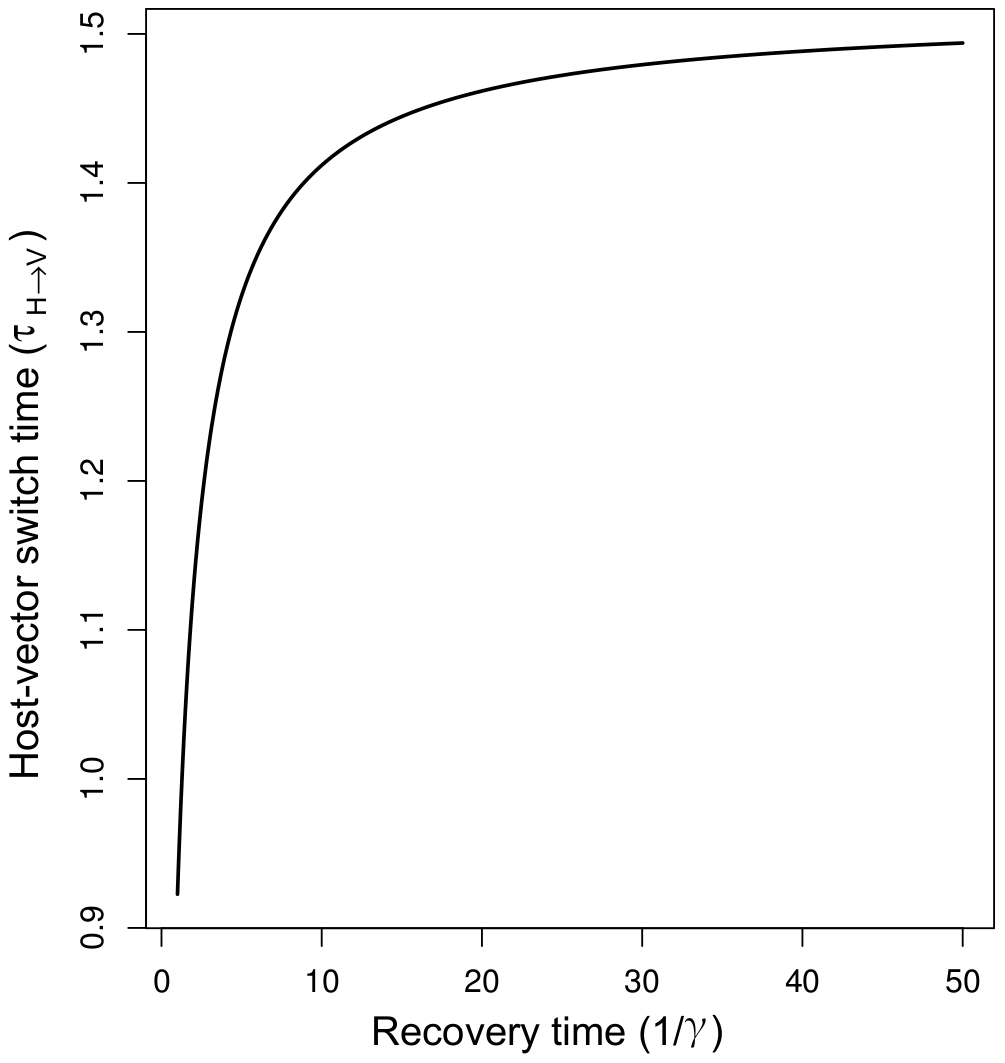
The critical time as a function of the unknown recovery time, 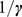. Because the switch time between infected hosts and vectors (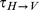) is always be less than two days, with weekly sampling periods it will be better to sample for infected vectors than for infected hosts.

Applying the linearized dynamics and binomial sampling model, we found that the critical switch between infected hosts and vectors, 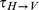 (equation (26)), was less than two days for all recovery times (Figure 3). Therefore, with a weekly sampling protocol it will be more economically efficient to sample for infected vectors rather than for infected hosts. We next tested whether it is better to sample infected vectors or recovered hosts. Looking at equation (28), we found it is optimal to sample from the recovered host class when 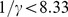. Otherwise, it is optimal to sample from the infected vectors class. Taken together, our analysis suggests that if the recovery time is less than about 8 days, it will be best to sample from the recovered host stratum; otherwise it will be best to sample from the infected vector stratum, and in all scenarios the infected host class can be ignored.

Applying equation (26) can potentially be misleading when the initial infection is presumed to be in the host population and it really originates in the vector. In this case the true switch time is given by 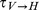 (Text S4, equation (S27)) for which a switch time does not exist for any 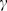. The switch from vectors to recovered hosts for these alternative initial conditions is given by equation (S27), and this exists when 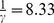. However, the switch times predicted are all very large with 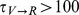 days so the optimal strategy would be to always sample for infected vectors. This suggests there would be error in this scenario from poor assumptions about the initial condition when 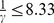, though for higher recovery times we correctly decide to sample infected vectors. We examine the potential costs of this incorrect decision in terms of the expected time to detection at the end of the following analysis.

**Figure 4:**
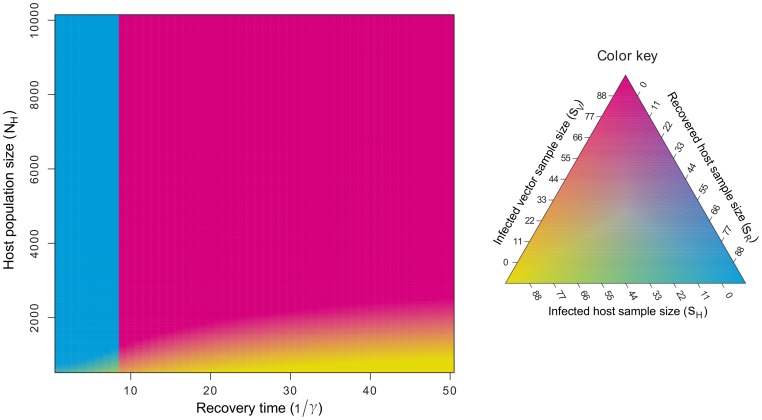
The optimal sampling strategy as a function of vector population abundance, 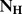 and the time to recovery, 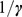. The pure magenta region corresponds to an optimal sampling design of 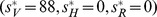, where the vector sample size is denoted as 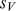, the sample size for testing infected hosts is denoted by 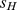, and the sample size for testing recovered hosts is denoted by 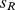. The pure cyan region corresponds to an optimal sampling design of 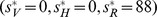. These two regions are separated at recovery times of 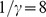. Regions with yellow correspond to mixed solutions of the form 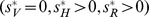 when 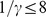, or 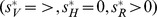 when 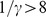.

We examined the robustness of our predictions using simulations of the full SIR model with geometric sampling to find the optimal sampling design 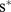 for recovery times ranging from 1 to 50 days (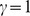 to 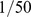). Our model predictions showed that 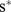 depends on the recovery time in a manner similar to the linearized solutions, however, at low 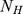 there was a narrow region of parameter space where a mixed solution was optimal. At sampling levels where the host sample size, 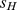, was 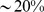 or more of the total host population size, 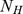, the optimal solution, 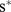, depended on the infected host population mixed with either recovered hosts (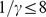) or infected vectors (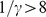) (Figure 4). The critical value of 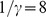 defined where the solution switches between recovered hosts and infected vectors consistent with predictions from the linearized model. There were no values of 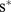 where all three classes were to be sampled. Overall, the linearized results provided a useful guide to the optimal sampling design except for the narrow region of parameter space where mixed sampling designs were found to be optimal at low 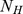 (Figure 4).

Our results from the linearized binomial sampling models for both the SI and SIR dynamics appeared robust to several of our assumptions as the effects of both the linearized dynamics and choice of sampling model had little effect on the optimal sampling design for West Nile virus. However, our results were sensitive to low host population sizes where the nonlinear models suggest that a mixed sampling design that incorporates both vectors and infected hosts will be optimal when it is possible to sample a significant proportion of the host population. In cases where the host population is sufficiently large, our analysis recommends placing sampling effort into infected vector populations given that current evidence suggests either long recovery times or persistent infections for West Nile virus in bird populations [30], [31]. When the sampling size is 20% or more of the total population size then more detailed models should be explored such as the full SI and SIR models.

**Figure 5:**
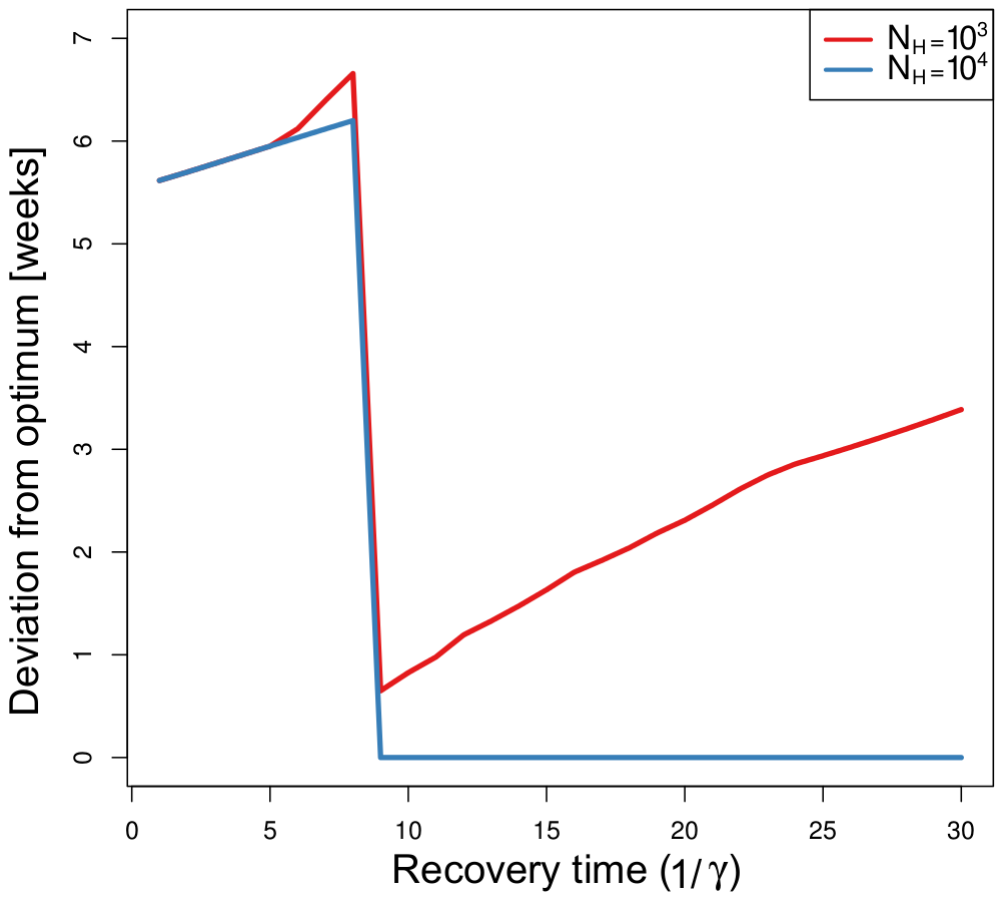
The sensitivity of the expected time to detection on initial conditions. We calculated the deviation from the minimal time to detection when we assume that an epidemic starts in the host population and the the number of initially infected vectors is zero (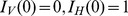), but the initial conditions of the outbreak are actually 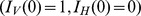. We did this for two host sample sizes of 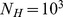 and 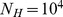, and over a range of recovery times 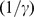 from 1 to 30 days.

We tested the sensitivity of the time to detection on the initial conditions by simulating the optimal decision of an outbreak with initial conditions 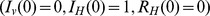 when the outbreak actually occurred with initial conditions 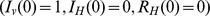 for 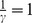 to 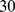 and 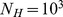 and 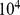. We found that when the recovery time, 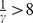, and 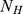 was high the optimal decisions between the two initial conditions were consistent and there was no error (Figure 5). However when 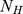 was low, or the recovery time was less than 8 days the optimal decisions differed strongly between initial conditions. This led to significant error in the expected time to detection, on the order of 5 to 6 weeks for low recovery times and 1–3 weeks for the low population sizes and high recovery times (Figure 5).

## Discussion

Active surveillance is an important tool for decision makers; treating the process analytically can provide some important insights on how to conduct cost-efficient surveillance. Very little past work in mathematical epidemiology has focused on early detection despite these potential benefits. One of the important products of this analysis has been to explicitly define the kinds of data that will be needed to design basic surveillance studies. Specific knowledge about the costs associated with sampling different populations and information about disease transmission rates will be necessary when making very specific predictions, but, as we have shown, applying the procedure with only basic knowledge of these quantities can make predictions that may be robust. This is fortunate for monitoring agencies as ecological and epidemiological parameters can be difficult and costly to obtain. Our analyses of West Nile virus illustrates robustness to parameters that are often unknown over a variety of models and assumptions.

Although we focused on basic SI and SIR models, this framework can be easily extended to include more specific models when they are available. It is likely that West Nile virus models that incorporate more biological realism (e.g., [32]) will be necessary to provide more targeted advice concerning surveillance practices for specific management agencies, whose monitoring capabilities may differ from what was assumed in this work. However, because the timescales we are examining are relatively short, our models may provide robust predictions when sampling is conducted over limited spatial scales. Therefore, even the simplified models examined here may be useful for designing sampling strategies when more detailed ecological and epidemiological information is not available.

Our results suggest that the optimal sampling design will often focus all sampling effort on a particular species or compartment. This result is due to the linear nature of the cost functions and the approximately linear nature of the dynamical systems as functions of our control variable, the sample sizes, 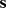. These on-off or “bang-bang” types of solutions arise in other epidemiological problems when determining how to treat or remove individuals in infected populations to stop an epidemic [33]–[35]. More recent work on the control of epidemics suggests that when considering multiple control strategies the optimal solution is not simply an additive combination of the independent control solutions [36]. Similar results may hold for surveillance methods when combining different types of surveillance strategies, for example active and passive sampling strategies. In cases where linearity and large population approximations for the dynamics do not hold, our analysis suggests that the optimal sampling design can be a mixture of sampling strata but this occurs over a very limited parameter space for West Nile virus (Figure 4). Nonlinear cost functions may also arise when the cost per sample changes when performing a large number of samples due to reductions in the associated personnel costs or in the laboratory fees incurred in performing a large number of tests. Changing the dynamical model by incorporating more detailed ecological and epidemiological considerations may also reduce the robustness of our linearization approximation. For example, introducing spatial structured populations [37] or heterogeneous contact rates are known to lead to additional nonlinearities in incidence functions [38], [39].

There are several additional considerations that may improve upon our efforts. Many disease models include exposed compartments (e.g., malaria [40]) in the host and/or vector population that can delay the onset of infectiousness once bitten. This may lead to additional possibilities in the switch time analysis that we did not consider. For example, if a host population is initially infected but has a long exposed period then there may be a quick switch to sampling the vector population followed by switches at longer time scales back to the host population. Additional important developments include treating the initial conditions and transmission process as random variables. This will likely lead to a distribution of optimal strategies rather than a single, fixed strategy [41]. Recognizing uncertainty in the initial conditions may be especially important when the source of infection is unclear given the potential sensitivity of the sampling process to the initial infections. We also did not consider the possibility of testing for multiple pathogens in this analysis. For example in Florida, mosquito control agencies regularly screen for malaria, West Nile virus, and dengue fever among others [12]. Applying a mixed sampling strategy may allow managers to hedge their bets because the optimal strategy for West Nile virus may not necessarily translate to the early detection of other pathogens. Finally, our assumption that diagnostic tests for pathogen or antibodies provide perfect indicators of an individual's state may be violated by several factors. First, immunological dynamics can lead to low viral or antibody levels even when individuals have been infected, which may lead to low test reliability [29]. Extending the approach to coupled immunological-epidemiological models may account for this source of uncertainty. Second, and perhaps more importantly, imperfect diagnostic test reliability can arise due to stochastic factors that cannot be accounted for in conventional lab techniques. These effects can be incorporated into a sampling model by multiplying the economic efficiency by a random variable representing the test sensitivity and specificity [42].

Despite the recognized impact of emerging zoonoses on human health [43] we are aware of no work that attempts to integrate the active surveillance systems explored here with disease surveillance in humans. In diseases where humans are spillover hosts, such as West Nile virus, low human incidence is expected. Passive surveillance is often more economically efficient when dealing with rare events [44] but this reporting process differs from the assumptions made in this work. In passive surveillence the reporting effort will often vary through time due to seasonal and institutional effects. Incorporating these factors into a predictive framework will require the statistical analyses of these patterns [11]. When including the surveillance of humans for West Nile virus we expect that reductions in the time to detection will occur when the recovery rate (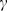) is high or the human population size is low relative the the vector population, as this is when hosts are most efficient to sample for detecting the disease (Figure 4), though the particular effects will depend on the amount of sampling effort and the transmission rate to humans from the vector. In general we expect that accounting for passive human surveillance of zoonoses may change the optimal active surveillance strategy for wildlife populations as it may not be necessary to sample hosts that have strong interactions with humans or species that significantly lag behind the epidemiological response of humans.

Another important case that we did not consider here are zoonotic diseases such as avian influenza, which spread much more easily within one zoologic species than across-species. For these diseases, the goal of surveillance is to detect a subtype of the disease more virulent in humans, indicated by sustained human to human transmission. This sampling needs to be tailored to detect clusters of human cases linked to a single avian-to-human transmission that deviate from what is to be expected from low-level human-to-human and bird-to-human transmission [45]. This kind of surveillance will require more detailed contact tracing that is not accounted for in our framework, though the basic structure we have described here could still be applied. More complex statistical analyses will also be needed to determine whether levels of infecteds and recovereds are significantly higher than background levels in order to determine if an outbreak is occurring. Analyses such as those determining epidemic thresholds from public health data (e.g. [45]–[47]) will be useful starting points for integrating thresholds into detecting epidemics of endemic zoonotic diseases.

## Supporting Information

Figure S1
**Vector field of system (S16) in the region** 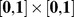 **.**
(TIFF)

Figure S2
**Possible solutions** 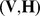 **of (S19).** The red dashed line is the line 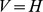.(TIFF)

Text S1 **General sampling probabilities.** Derivation of sampling probabilities for more than two strata.
(PDF)

Text S2 **KKT conditions.** Application of the Karush-Kuhn-Tucker conditions to the binomial sampling model with general constraints.
(PDF)

Text S3 **General SI analysis.** Derivation of general solutions for the SI model with binomial sampling.
(PDF)

Text S4 **Critical time for alternative initial conditions.** Derivation of critical times for when the initial infected individual is in the vector population.
(PDF)
