# Supplementary figures and images for: Optimal Sampling Strategies for Detecting Zoonotic Disease Epidemics
Source: PLoS Comput Biol. 2014 Jun 26;10(6):e1003668. doi: 10.1371/journal.pcbi.1003668 (PMC4072525; doi:10.1371/journal.pcbi.1003668)

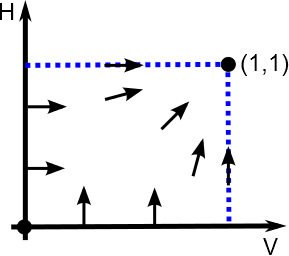

Supplement: Figure S1 — Vector field of system (S16) in the region . (TIFF) [file pcbi.1003668.s001.tiff]

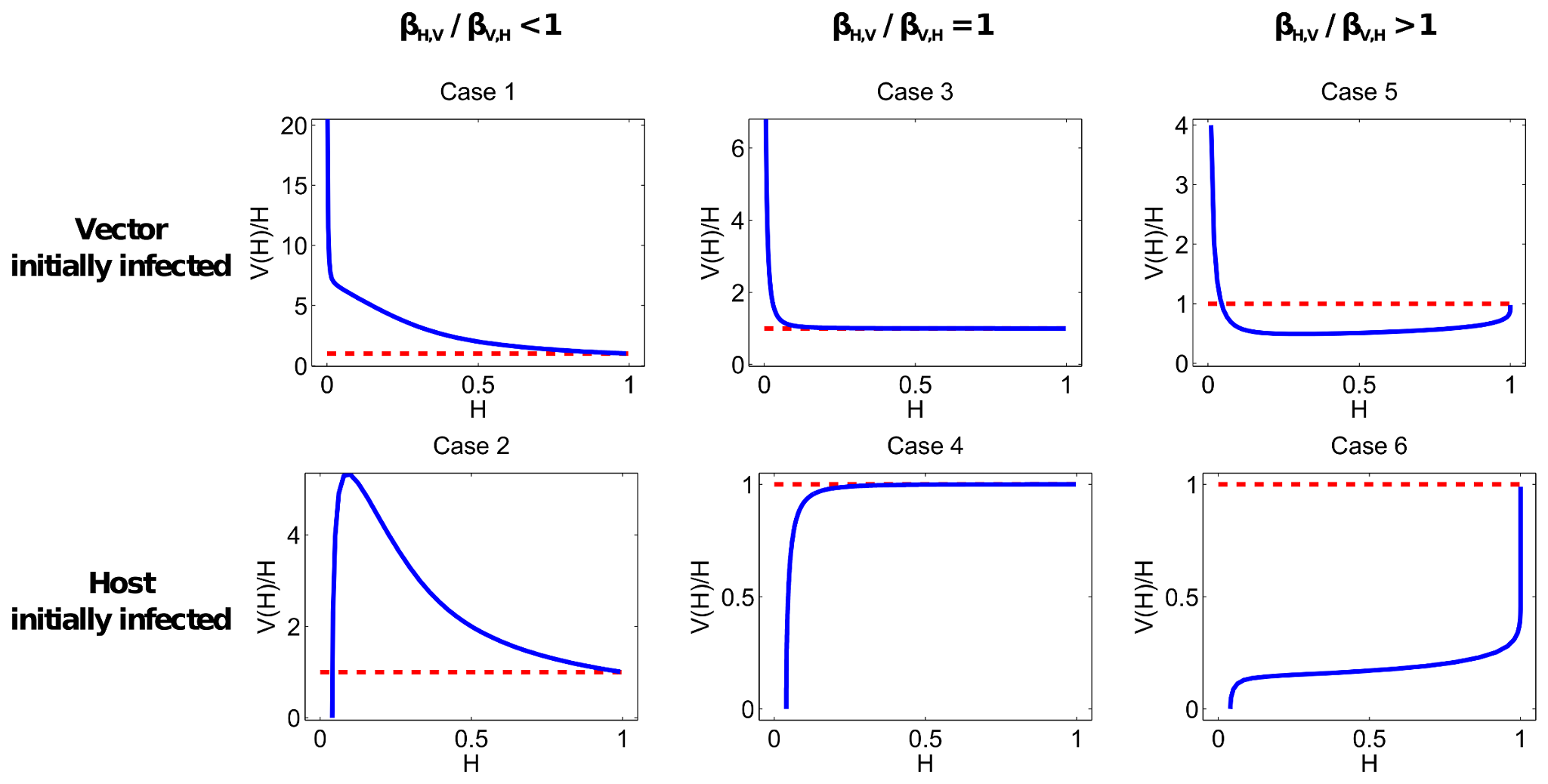

Supplement: Figure S2 — Possible solutions of (S19). The red dashed line is the line . (TIFF) [file pcbi.1003668.s002.tiff]
