# Supplementary material for: Optimal Sampling Strategies for Detecting Zoonotic Disease Epidemics
Source: PLoS Comput Biol. 2014 Jun 26;10(6):e1003668. doi: 10.1371/journal.pcbi.1003668 (PMC4072525; doi:10.1371/journal.pcbi.1003668)
Supplement: Text S1 — General sampling probabilities. Derivation of sampling probabilities for more than two strata. (PDF) [file pcbi.1003668.s003.pdf]

S1 General sampling probabilities

The general expression for n strata is given by

$$\begin{aligned} P\left(\bigcup_{i=1}^{n} D_i(t)\right) &= P\left(\left(\bigcup_{i=1}^{n-1} D_i(t)\right) \cup D_n(t)\right) \\ &= P\left(\bigcup_{i=1}^{n-1} D_i(t)\right) + P\left(D_n(t)\right) - P\left(\left(\bigcup_{i=1}^{n-1} D_i(t)\right) \cap D_n(t)\right). \end{aligned} \tag{S1}$$

We claim that

$$P\left(\bigcup_{i=1}^{n} D_i(t)\right) = 1 - e^{-\sum_{i=1}^{n} s_i I_i(t)/N_i} \tag{S2}$$

for all $n \geq 1$. We prove this claim by induction. First note that the $n = 1$ case is due to the binomial expansion of equation (7) and that the $n = 2$ case holds by (9). Suppose that (S2) holds for $n - 1$, that is, that for $n - 1$ the probability model is given by

$$P\left(\bigcup_{i=1}^{n-1} D_i(t)\right) = 1 - e^{-\sum_{i=1}^{n-1} s_i I_i(t)/N_i}.$$

We will show that (S2) holds for n. Substituting the expression above into (S1), the formula for the n species case, we have

$$\begin{aligned} P\left(\bigcup_{i=1}^{n} D_i(t)\right) &= P\left(\bigcup_{i=1}^{n-1} D_i(t)\right) + P\left(D_n(t)\right) - P\left(\left(\bigcup_{i=1}^{n-1} D_i(t)\right) \cap D_n(t)\right) \\ &= (1 - e^{-\sum_{i=1}^{n-1} s_i I_i(t)/N_i}) + (1 - e^{-s_n I_n(t)/N_n}) \\ &\quad - (1 - e^{-\sum_{i=1}^{n-1} s_i I_i(t)/N_i})(1 - e^{-s_n I_n(t)/N_n}) \\ &= (2 - e^{-\sum_{i=1}^{n-1} s_i I_i(t)/N_i} - e^{-s_n I_n(t)/N_n}) \\ &\quad - (1 - e^{-\sum_{i=1}^{n-1} s_i I_i(t)/N_i} - e^{-s_n I_n(t)/N_n} + e^{-\sum_{i=1}^{n} s_n I_n(t)/N_n}) \\ &= 1 - e^{-\sum_{i=1}^{n} s_i I_i(t)/N_i}. \end{aligned}$$

Thus, equation (S2) holds for any n species and our claim is proven.
