# Supplementary material for: Optimal Sampling Strategies for Detecting Zoonotic Disease Epidemics
Source: PLoS Comput Biol. 2014 Jun 26;10(6):e1003668. doi: 10.1371/journal.pcbi.1003668 (PMC4072525; doi:10.1371/journal.pcbi.1003668)
Supplement: Text S2 — KKT conditions. Application of the Karush-Kuhn-Tucker conditions to the binomial sampling model with general constraints. (PDF) [file pcbi.1003668.s004.pdf]

S2 KKT conditions

In this appendix, we will apply the Karush-Kuhn-Tucker optimization conditions to the probability model (10) derived in section S1. The probability of detecting a disease which can infect n populations is given by

$$P\left(\bigcup_{i=1}^{n} D_i(t)\right) = 1 - e^{-\sum_{i=1}^{n} s_i I_i(t)/N_i}$$

for all $n \geq 1$ where s_i is the number of samples taken from population i, $1 \leq i \leq n$, and D_i is the set of events such that the disease is detected from a sample of size s_i from population i.

To maximize the probability of detecting at least one infected individual, we must minimize

$$f(s_1, \dots s_n) := -\sum_{i=1}^{n} s_i \frac{I_i(t)}{N_i}. \tag{S3}$$

Though completely unrealistic, the above quantity would be minimized if we take s_i, the number of sampled individuals, arbitrarily large. To incorporate a modicum of realism, we assume that we must sample under a given cost constraint. We let $C_{\max}$ be the budget for a given sampling scheme $s = (s_1, \dots s_n)$ and let $C(s_1, \dots s_n)$ be the cost of sampling s_i individuals from population i. If we assume that we spend our entire budget, we have introduced the cost constraint $C(s_1, \dots s_n) = C_{\max}$. We additionally require that all sample sizes s_i are nonnegative.

To minimize the objective function (S3) under the constraints

$$h(s) := C(s_1, \dots s_n) - C_{\max} = 0 \tag{S4}$$

$$g(s) := \begin{pmatrix} -s_1 \\ \vdots \\ -s_n \end{pmatrix} \leq 0 \tag{S5}$$

where $s \in \mathbb{R}^n$, we apply the Karush-Kuhn-Tucker conditions. If $s^* \in \mathbb{R}^n$ is a local minimum of the objective function (S3), then there exist constants μ_i, $1 \leq i \leq n$ and λ such that

$$\nabla f(s^*) + \sum_{i=1}^{n} \mu_i \nabla g_i(s^*) + \lambda \nabla h(s^*) = 0 \tag{S6}$$

$$\mu_i \geq 0,\ \ 1 \leq i \leq n \tag{S7}$$

$$\mu_i g_i(s^*) = 0,\ \ 1 \leq i \leq n \tag{S8}$$

where f, g and h are defined in (S3), (S4) and (S5).

KKT Conditions for a linear objective function

Since our objective function $f(s)$ and primal feasibility condition (S5) are linear, the stationarity equation (S6) is relatively simple:

$$\nabla f(s^*) + \sum_{i=1}^{n} \mu_i \nabla g_i(s^*) + \lambda \nabla h(s^*) = 0$$

$$\begin{pmatrix} -\frac{I_1(t)}{N_1} \\ \vdots \\ -\frac{I_n(t)}{N_n} \end{pmatrix} + \begin{pmatrix} -\mu_1 \\ \vdots \\ -\mu_n \end{pmatrix} + \lambda \begin{pmatrix} C_{s_1}(s^*) \\ \vdots \\ C_{s_n}(s^*) \end{pmatrix} = 0$$

where $C_{s_i}(s^*) = \frac{\partial C}{\partial s_i}(s^*)$. If we let $P_i(t) = \frac{I_i(t)}{N_i}$ be the proportion of individuals in population i that are infected at time t, the above expression becomes

$$\begin{pmatrix} -P_1(t) \\ \vdots \\ -P_n(t) \end{pmatrix} + \begin{pmatrix} -\mu_1 \\ \vdots \\ -\mu_n \end{pmatrix} + \lambda \begin{pmatrix} C_{s_1}(s^*) \\ \vdots \\ C_{s_n}(s^*) \end{pmatrix} = 0. \tag{S9}$$

The dual feasibility (S7) and complementary slackness (S8) conditions become

$$\mu_i \geq 0, \;\; 1 \leq i \leq n \tag{S10}$$
$$\mu_i s_i^* = 0, \;\; 1 \leq i \leq n. \tag{S11}$$

In the following analysis, we assume that $C_{s_i}(s) > 0$ for each i and all nonnegative s, that is, we assume that increasing the number of samples increases the cost of sampling. To find candidates for the local minimizer s^*, we solve (S9) in four cases. A summary of these four cases is given in Table 1 in Text S2.

Table 1 in Text S2. Summary of Section S2 with all possible cases listed.

	λ	μ_i	$P_i(t)$	s^*
Case 1	$\lambda = 0$	$\mu_i = 0$, $1 \leq i \leq n$	$P_i(t) = 0$, $1 \leq i \leq n$	$s^* \in \mathbb{R}^n_+$
Case 2	$\lambda \neq 0$<br>$\frac{P_i(t)}{C_{s_i}(s^*)} = \lambda$<br>$1 \leq i \leq n$	$\mu_i = 0$, $1 \leq i \leq n$	$P_i(t) > 0$, $1 \leq i \leq n$	$s^* \in \mathbb{R}^n_+$
Case 3	$\lambda \neq 0$	$\mu_i \neq 0$, $1 \leq i \leq n$	$P_i(t) \geq 0$, $1 \leq i \leq n$	$s^* = \vec{0}$
Case 4	$\lambda \neq 0$<br>$\frac{P_i(t)}{C_{s_i}(s^*)} = \lambda$<br>$1 \leq i \leq k$	$\mu_i = 0$, $1 \leq i \leq k$<br>$\mu_j \neq 0$, $k+1 \leq j \leq n$	$P_i(t) > 0$, $1 \leq i \leq k$<br>$\frac{P_i(t)}{C_{s_i}(s^*)} > \frac{P_j(t)}{C_{s_j}(s^*)}$<br>$k+1 \leq j \leq n$	$s_i^* \geq 0$, $1 \leq i \leq k$<br>$s_j^* = 0$, $k+1 \leq j \leq n$

Case 1: $\lambda = 0$.
If $\lambda = 0$, then (S9) becomes

$$\begin{pmatrix} -P_1(t) \\ \vdots \\ -P_n(t) \end{pmatrix} + \begin{pmatrix} -\mu_1 \\ \vdots \\ -\mu_n \end{pmatrix} = 0$$
$$\begin{pmatrix} -P_1(t) \\ \vdots \\ -P_n(t) \end{pmatrix} = \begin{pmatrix} \mu_1 \\ \vdots \\ \mu_n \end{pmatrix}$$

By (S10) and since $P_i(t) \geq 0$, we have that $\lambda = 0$ if and only if $P_i(t) = 0$ for all $1 \leq i \leq n$. (Indeed, suppose that $P_i(t) > 0$ for some $1 \leq i \leq n$. Then $\mu_i = -P_i(t) < 0$, a contradiction to (S10).) Then it must also be true that $\mu_i = 0$ for all $1 \leq i \leq n$. Then (S11) is satisfied for any choice of s_i^*. This implies that if the disease is not present, any sampling scheme will give the same (zero) probability of detection.
Case 2: $\lambda \neq 0$, $\mu_i = 0$, $1 \leq i \leq n$.
If $\mu_i = 0$, $1 \leq i \leq n$, then (S9) becomes

$$\lambda \begin{pmatrix} C_{s_1}(s^*) \\ \vdots \\ C_{s_n}(s^*) \end{pmatrix} = \begin{pmatrix} P_1(t) \\ \vdots \\ P_n(t) \end{pmatrix}.$$

Since $C_{s_i}(s) > 0$ for all nonnegative s and all i,

$$\lambda = \frac{P_i(t)}{C_{s_i}(s^*)}$$

for all i. Then

$$\frac{P_i(t)}{C_{s_i}(s^*)} = \lambda = \frac{P_j(t)}{C_{s_j}(s^*)} \quad \text{for all } 1 \le i \le n, 1 \le j \le n. \tag{S12}$$

Since $\mu_i = 0$, $1 \le i \le n$, (S11) holds for any choice of s_i^*, $1 \le i \le n$. Note that $\lambda \neq 0$ then implies that $P_i(t) > 0$ for all i. Thus, the above analysis implies that it is possible that the optimal sampling scheme is to sample all of the populations if and only if $P_i > 0$ for all $1 \le i \le n$. Indeed, otherwise (S12) implies that $0 = \lambda = P_i$ for all $1 \le i \le n$.

<u>Case 3: $\lambda \neq 0$, $\mu_i \neq 0$, $1 \le i \le n$.</u>

By (S11), if $\mu_i \neq 0$, then $s_i^* = 0$. Since $\mu_i \neq 0$ for all $1 \le i \le n$, this implies that $s^* = \vec{0}$. Since s^* must satisfy (S4), this corresponds to the case where the total overhead cost equals the budget: $C(0) = C_{max}$.

<u>Case 4: $\lambda \neq 0$, $\mu_i = 0$, $1 \le i \le k$, $\mu_j \neq 0$, $k+1 \le i \le n$ for some integer $k \in (1, n)$.</u>

Fix some integer $k \in (1, n)$. Suppose that $\mu_i = 0$ for all $1 \le i \le k$ and $\mu_j \neq 0$ for all $k+1 \le i \le n$. Then, as in Case 3, (S11) implies that $s_j^* = 0$ for $k+1 \le j \le n$ and (S9) becomes

$$\begin{pmatrix} -P_1(t) \\ \vdots \\ -P_k(t) \\ -P_{k+1}(t) \\ \vdots \\ -P_n(t) \end{pmatrix} + \begin{pmatrix} 0 \\ \vdots \\ 0 \\ -\mu_{k+1} \\ \vdots \\ -\mu_n \end{pmatrix} + \lambda \begin{pmatrix} C_{s_1}(s^*) \\ \vdots \\ C_{s_k}(s^*) \\ C_{s_{k+1}}(s^*) \\ \vdots \\ C_{s_n}(s^*) \end{pmatrix} = 0. \tag{S13}$$

As in Case 2, the first k equations above imply that

$$\frac{P_i(t)}{C_{s_i}(s^*)} = \lambda = \frac{P_j(t)}{C_{s_j}(s^*)} \quad \text{for all } 1 \le i \le k, 1 \le j \le k \tag{S14}$$

since $C_{s_i}(s) > 0$ for all nonnegative s. Then, since $\lambda > 0$,

$$P_i(t) > 0 \text{ for all } 1 \le i \le k.$$

Furthermore, the last $n - k$ equations of (S13) together with (S14) imply that

$$\begin{aligned} -P_j(t) - \mu_j + \lambda C_{s_j}(s^*) &= 0, \;\; k+1 \le j \le n \\ -P_j(t) - \mu_j + \frac{P_i(t)}{C_{s_i}(s^*)} C_{s_j}(s^*) &= 0, \;\; 1 \le i \le k, k+1 \le j \le n \\ -P_j(t) + \frac{P_i(t)}{C_{s_i}(s^*)} C_{s_j}(s^*) &= \mu_j > 0, \;\; 1 \le i \le k, k+1 \le j \le n \\ \iff \qquad \frac{P_i(t)}{C_{s_i}(s^*)} &> \frac{P_j(t)}{C_{s_j}(s^*)}, \;\; 1 \le i \le k, k+1 \le j \le n \end{aligned} \tag{S15}$$

by (S10) since $\mu_j \neq 0$ for $k+1 \leq j \leq n$. Thus, the optimal sampling scheme may be to sample the first k populations if and only if (S15) holds.
