# Supplementary material for: Optimal Sampling Strategies for Detecting Zoonotic Disease Epidemics
Source: PLoS Comput Biol. 2014 Jun 26;10(6):e1003668. doi: 10.1371/journal.pcbi.1003668 (PMC4072525; doi:10.1371/journal.pcbi.1003668)
Supplement: Text S3 — General SI analysis. Derivation of general solutions for the SI model with binomial sampling. (PDF) [file pcbi.1003668.s005.pdf]

S3 General SI Analysis

We now give an example of how the analysis in section S2 can be used to determine the optimal sampling scheme for maximizing the probability of detecting a vector borne disease that follows SI dynamics. In the following, we use system (1) (presented in the main text) to describe the dynamics of a disease. We consider the case where there is one vector population and one host population. Thus we consider the system

$$\frac{d}{dt}I_V(t) = \beta_{V,H}\frac{I_H(t)}{N_H}\left(N_V - I_V(t)\right)$$
$$\frac{d}{dt}I_H(t) = \beta_{H,V}\frac{I_V(t)}{N_V}\left(N_H - I_H(t)\right)$$

where we denote the number of infected vectors by $I_V(t)$, the number of infected hosts by $I_H(t)$, the total number of vectors by N_V, the total number of hosts by N_H, the transmission rate from hosts to vectors by $\beta_{V,H}$, and the transmission rate from vectors to hosts by $\beta_{H,V}$.

For ease of notation, define

$$V(t) = \frac{I_V(t)}{N_V} \quad \text{and} \quad H(t) = \frac{I_H(t)}{N_H}$$

to be the proportion of the infected vector (host) population. Then rewriting the above system, we have that

$$\begin{aligned}
\frac{d}{dt}I_V(t) &= \beta_{V,H}\frac{I_H(t)}{N_H}S_v(t)\\
&= \beta_{V,H}\frac{I_H(t)}{N_H}(N_V - I_V(t))\\
\frac{d}{dt}\frac{I_V(t)}{N_V} &= \beta_{V,H}\frac{I_H(t)}{N_H}\left(1 - \frac{I_V(t)}{N_V}\right)\\
\frac{d}{dt}V(t) &= \beta_{V,H}H(t)(1 - V(t))
\end{aligned}$$

Similarly,

$$\frac{d}{dt}H(t) = \beta_{H,V}V(t)(1 - H(t)).$$

Lastly, for ease of notation, redefine

$$\beta_{V,H} = \alpha \quad \text{and} \quad \beta_{H,V} = \gamma.$$

Then our system becomes

$$\frac{d}{dt}V(t) = \alpha H(t)(1 - V(t)) \tag{S16a}$$
$$\frac{d}{dt}H(t) = \gamma V(t)(1 - H(t)) \tag{S16b}$$

The model variables and parameters are summarized in Table 2 in Text S3.

Table 2 in Text S3. SI model parameters and variables.

Parameter or Variable	Definition
I_H	Number of infected hosts.
S_h	Number of susceptible hosts.
I_V	Number of infected vectors.
S_v	Number of susceptible vectors.
N_H	Total number of hosts.
N_V	Total number of vectors.
$H = \frac{I_H}{N_H}$	Proportion of infected hosts.
$V = \frac{I_V}{N_V}$	Proportion of infected vectors.
$\beta_{V,H} = \alpha$	Transmission rate from hosts to vectors.
$\beta_{H,V} = \gamma$	Transmission rate from vectors to hosts.

S3.1 Basic analysis of (S16)

It is easy to see that system (S16) has two steady states, $(V, H) = (0, 0)$ and $(1, 1)$. Examining the vector field of (S16), we see that

$$
\begin{aligned}
V = 0,\ 0 < H < 1 \Rightarrow \quad & \frac{dV}{dt} = \alpha H > 0, && \frac{dH}{dt} = 0 \\
0 < V < 1,\ H = 0 \Rightarrow \quad & \frac{dV}{dt} = 0, && \frac{dH}{dt} = \gamma V > 0 \\
0 < V < 1,\ H = 1 \Rightarrow \quad & \frac{dV}{dt} = \alpha(1 - V) > 0, && \frac{dH}{dt} = 0 \\
V = 1,\ 0 < H < 1 \Rightarrow \quad & \frac{dV}{dt} = 0, && \frac{dH}{dt} = \gamma(1 - H) > 0 \\
0 < V < 1,\ 0 < H < 1 \Rightarrow \quad & \frac{dV}{dt} = \alpha H(1 - V) > 0, && \frac{dH}{dt} = \gamma V(1 - H) > 0
\end{aligned}
$$

Thus, given an initial condition (V_0, H_0) such that $V_0, H_0 \in [0, 1]$, $(V_0, H_0) \notin \{(0, 0), (1, 1)\}$, the solution $(V(t), H(t))$ approaches $(1, 1)$ in infinite time. (Figure S1) Though this is not a realistic scenario, we are only concerned with the early-time behavior of the system and the infinite time dynamics are only of academic interest.

S3.2 First integral

Note that if $V(t) > 0$, then $\frac{dH}{dt} > 0$ for all $0 \leq H(t) < 1$. Similarly, if $H(t) > 0$, then $\frac{dV}{dt} > 0$ for all $0 \leq V(t) < 1$. Note that neither V nor H may become negative so long as $V(0) \geq 0$ and $H(0) \geq 0$. Then we may reparameterize our system (S16) as a function of V or as a function of H. We will choose to reparameterize our system as a function of H. Dividing (S16a) by (S16b), we have the auxiliary equation

$$
\begin{aligned}
\frac{dV}{dH} &= \frac{\alpha H(t)(1 - V(t))}{\gamma V(t)(1 - H(t))} \\
&= \frac{\frac{\alpha}{V} - \alpha}{\frac{\gamma}{H} - \gamma}
\end{aligned}
\tag{S17}
$$

which we can solve by separation of variables:

$$dV\left(\frac{V}{\alpha-\alpha V}\right) = dH\left(\frac{H}{\gamma-\gamma H}\right)$$
$$\int\left(\frac{V}{\alpha-\alpha V}\right)dV = \int\left(\frac{H}{\gamma-\gamma H}\right)dH$$
$$\frac{1}{\alpha}\left(-V-\ln(1-V)\right) = \frac{1}{\gamma}\left(-H-\ln(1-H)\right)+c$$

where c is some constant. Then solutions of (S16) lie within the level sets of the function

$$I(V,H) = \frac{1}{\alpha}\left(-V-\ln(1-V)\right) + \frac{1}{\gamma}\left(H+\ln(1-H)\right).$$

In particular, given an initial condition (V_0, H_0), the solution (V,H) of the initial value problem (S16), $V(0) = V_0$, $H(0) = H_0$ satisfies

$$I(V,H) = I(V_0,H_0)$$
$$\frac{1}{\alpha}\left(-V-\ln(1-V)\right) + \frac{1}{\gamma}\left(H+\ln(1-H)\right) = \frac{1}{\alpha}\left(-V_0-\ln(1-V_0)\right) + \frac{1}{\gamma}\left(H_0+\ln(1-H_0)\right)$$

that is, the solution (V,H) lies in the level set

$$\{(V,H)|I(V,H) = I(V_0,H_0)\}.$$

S3.3 Optimal sampling

Suppose that $C(s_V, s_H)$ is a strictly increasing cost function where s_V denotes the number of vectors sampled and s_H denotes the number of hosts sampled. Our goal is to find possible optimal sampling schemes $s^* = (s_V^*, s_H^*)$ that maximize the probability of detecting a disease in a single sampling trial at a fixed time t, assuming that the vector and host population dynamics are known. With reference to Table 1 in Text S2, we see that there are three possible sampling schemes. First (Case 2 in Table 1 in Text S2), if there exists some s^* such that $s_V^* \geq 0$ and $s_H^* \geq 0$ and

$$\frac{V(t)}{C_{s_V}(s^*)} = \frac{H(t)}{C_{s_H}(s^*)}$$
$$\iff \quad \frac{V(t)}{H(t)} = \frac{C_{s_V}(s^*)}{C_{s_H}(s^*)}$$

then we may choose to sample both the vector and the host populations. Second (Case 4 in Table 1 in Text S2), if there exists some s^* such that $s_V^* \geq 0$ and $s_H^* = 0$ and

$$\frac{V(t)}{C_{s_V}(s^*)} > \frac{H(t)}{C_{s_H}(s^*)}$$
$$\iff \quad \frac{V(t)}{H(t)} > \frac{C_{s_V}(s^*)}{C_{s_H}(s^*)}$$

then we may choose to sample only the vector population. Third (Case 4 in Table 1 in Text S2), if there exists some s^* such that $s_V^* = 0$ and $s_H^* \geq 0$ and

$$\frac{V(t)}{C_{s_V}(s^*)} < \frac{H(t)}{C_{s_H}(s^*)}$$
$$\iff \quad \frac{V(t)}{H(t)} < \frac{C_{s_V}(s^*)}{C_{s_H}(s^*)}$$

then we may choose to sample only the host population. Since each of these cases depends on the ratio $\frac{V}{H}$, we now characterize this curve. We will first restate two useful equations and make some easy observations. Then, we will give two lemmas that elucidate some properties of the curve $\frac{V(H)}{H}$.

First, by (S17),

$$\frac{dV}{dH} = \frac{\alpha H (1-V)}{\gamma V (1-H)} \tag{S18}$$

Then $\frac{dV}{dH} > 0$ for all $(V, H) \in [0,1] \times [0,1] \backslash \{(0,0), (1,1)\}$. Since $V(H)$ is increasing and $V(H) \leq 1$ for $H \in (0,1)$, $\lim_{H \to 1} V(H)$ exists. We claim that for $(V_0, H_0) \in [0,1) \times [0,1) \backslash \{(0,0)\}$, $\lim_{H \to 1} V(H) = 1$. If not, then there exists some $0 < M < 1$ such that $\lim_{H \to 1} V(H) = M$. Then by the analysis in Section S3.1, it is easy to see that $H_0 = 1$, a contradiction to the uniqueness of solutions.

Suppose that the initial condition (V_0, H_0) is given, suppose that only one of V_0 or H_0 is positive, and let (V, H) be the solution to this initial value problem. Let $I(V_0, H_0) = c$. Then recall that (V, H) solves

$$\frac{1}{\alpha}(-V - \ln(1-V)) + \frac{1}{\gamma}(H + \ln(1-H)) = c. \tag{S19}$$

Note that

$$\begin{aligned} V_0 = 0, H_0 > 0 &\Rightarrow I(V_0, H_0) < 0 \\ V_0 = 0, H_0 = 0 &\Rightarrow I(V_0, H_0) = 0 \\ V_0 > 0, H_0 = 0 &\Rightarrow I(V_0, H_0) > 0 \end{aligned}$$

by (S19). Then, since at most one of V_0 or H_0 is positive, any initial condition (V_0, H_0) must satisfy exactly one of the above conditions. Since the sign of $I(V_0, H_0)$ implied in the above relations is unique for each class of initial condition (V_0, H_0), we have that

$$\begin{aligned} V_0 = 0, H_0 > 0 &\Leftarrow I(V_0, H_0) < 0 \\ V_0 = 0, H_0 = 0 &\Leftarrow I(V_0, H_0) = 0 \\ V_0 > 0, H_0 = 0 &\Leftarrow I(V_0, H_0) > 0. \end{aligned}$$

We now prove two lemmas that are useful in characterizing the curve $\frac{V(H)}{H}$.

Lemma 1. *Under the following conditions, there exists some unique $H^* \in (H_0, 1)$ that solves $V(H) = H$:*

1. *$\alpha < \gamma$ and $I(V_0, H_0) = c > 0$ or*
2. *$\alpha > \gamma$ and $I(V_0, H_0) = c < 0$.*

Otherwise, there exists no such H^*.

Proof. Suppose that

$$\left(\frac{1}{\alpha} - \frac{1}{\gamma}\right)(-H^* - \ln(1-H^*)) = c. \tag{S20}$$

Then by (S19),

$$-H^* - \ln(1-H^*) = -V(H^*) - \ln(1 - V(H^*)).$$

Since the function $F(x) = -x - \ln(1-x)$ is strictly increasing for $x \in (0,1)$, the above equation implies that $V(H^*) = H^*$. We now show that there exists some $H^* \in (0,1)$ that solves (S20).

Note that the function $F(x) = -x - \ln(1-x)$ is positive and strictly increasing for $x \in (0,1)$. In addition, $F(0) = 0$ and $\lim_{H \to 1} F(H) = \infty$. Then, since c, α and γ are constants, there exists

some unique $H^* \in (0,1)$ that solves (S20) if and only if $c \neq 0$ and the sign of c is the same as the sign of $\left(\frac{1}{\alpha} - \frac{1}{\gamma}\right)$. It remains only to be shown that $H^* \in (H_0, 1)$.

If Condition (1) in Lemma 1 holds, then $H_0 = 0$ and $H^* \in (H_0, 1) = (0,1)$ trivially. If Condition (2) in Lemma 1 holds, then $H_0 > 0$, $V_0 = 0$,

$$0 > \left(\frac{1}{\alpha} - \frac{1}{\gamma}\right)(-H_0 - \ln(1 - H_0)) > -\frac{1}{\gamma}(-H_0 - \ln(1 - H_0)) = c$$

by (S19) and

$$\lim_{H \to 1} \left(\frac{1}{\alpha} - \frac{1}{\gamma}\right)(-H - \ln(1 - H)) = -\infty.$$

Then there exists some $H^* \in (H_0, 1)$ which solves (S20). □

Lemma 1 gives conditions under which the curve $\frac{V(H)}{H}$ intersects the horizontal line at 1. Note that if there exists some $H^* \in (H_0, 1)$ such that $V(H^*) = H^*$, then $\frac{V(H^*)}{H^*} = 1$. If no such H^* exists, then the curve $\frac{V(H)}{H}$ must remain above or below the horizontal line at 1 for all $H \in (H_0, 1)$.

Lemma 2. *Suppose that $H < V(H)$ for all $H \in (H_1, H_2) \subseteq (H_0, 1)$, $\alpha > \gamma > 0$. Then there exists at most one $\bar{H} \in (H_1, H_2)$ such that $\left.\frac{d}{dH}\frac{V(H)}{H}\right|_{H=\bar{H}} = 0$.*

Proof. Note that

$$\begin{aligned} \frac{d}{dH}\frac{V(H)}{H} &= \frac{H\frac{dV}{dH} - V}{H^2} \\ &= \frac{\alpha H\left(\frac{1}{V} - 1\right) - \gamma V\left(\frac{1}{H} - 1\right)}{\gamma H(1 - H)} \end{aligned} \tag{S21}$$

and suppose $\bar{H} \in (H_1, H_2)$ such that $\left.\frac{d}{dH}\frac{V(H)}{H}\right|_{H=\bar{H}} = 0$. Since the denominator of (S21) is strictly positive, it must be true that

$$\begin{aligned} \alpha\bar{H}\left(\frac{1}{V(\bar{H})} - 1\right) &= \gamma V(\bar{H})\left(\frac{1}{\bar{H}} - 1\right) \\ \alpha\left(\frac{1 - V(\bar{H})}{V^2(\bar{H})}\right) &= \gamma\left(\frac{1 - \bar{H}}{\bar{H}^2}\right). \end{aligned} \tag{S22}$$

Now,

$$\begin{aligned} \frac{d}{dH}\frac{1 - V(\bar{H})}{V^2(\bar{H})} &= \frac{1}{V^4}\left[-V^2\frac{dV}{dH} - 2V(1 - V)\frac{dV}{dH}\right] \\ &= \frac{\alpha(V - 2)H(1 - V)}{\gamma V^4(1 - H)} \end{aligned}$$

by (S18) and

$$\frac{d}{dH}\frac{1 - H}{H^2} = \frac{H - 2}{H^3}.$$

We claim that

$$\frac{d}{dH}\frac{1 - V(\bar{H})}{V^2(\bar{H})} > \frac{d}{dH}\frac{1 - H}{H^2} \tag{S23}$$

for $H \in (H_1, H_2)$. (S23) holds if and only if

$$\frac{\alpha(V-2)H(1-V)}{\gamma V^4(1-H)} > \frac{H-2}{H^3}$$
$$\frac{\alpha(V-2)(1-V)}{V^4} > \frac{\gamma(H-2)(1-H)}{H^4}.$$

Since $\frac{(x-2)(1-x)}{x^4}$ is an increasing function for $x \in [0,1]$ and since $H < V$ we have that

$$\frac{(V-2)(1-V)}{V^4} > \frac{(H-2)(1-H)}{H^4}$$
$$\frac{\alpha(V-2)(1-V)}{V^4} > \frac{\gamma(H-2)(1-H)}{H^4}$$

since $\alpha > \gamma$. Thus (S23) holds. Since $\alpha > \gamma > 0$,

$$\frac{d}{dH}\alpha\left(\frac{1-V(\bar{H})}{V^2(\bar{H})}\right) > \frac{d}{dH}\gamma\left(\frac{1-H}{H^2}\right)$$

for all $H \in (H_1, H_2)$. Then if there exists some $\bar{H} \in (H_1, H_2) \subseteq (0,1)$ such that (S22) holds, it is unique. □

Assuming that the disease starts in either the vector population or the host population (not both), there are six possible characterizations of $\frac{V(H)}{H}$:

<u>Case 1: $\alpha > \gamma$, $V_0 > 0$, $H_0 = 0$.</u> Since $V_0 > 0$ and $H_0 = 0$, we have that $c > 0$. Note that $\frac{V(H_0)}{H_0} = \frac{V_0}{H_0} = \infty$. Then since $\alpha > \gamma$, $\frac{V(H)}{H} > 1$ for all $H \in (H_0, 1)$ by Lemma 1.

We claim that $\frac{d}{dH}\frac{V(H)}{H} \leq 0$ for all $H \in (H_0, 1)$. Indeed, if not, then there exists some $\tilde{H} \in (H_0, 1)$ such that $\frac{d}{dH}\frac{V(H)}{H}\Big|_{H=\tilde{H}} > 0$. Note that since $V_0 > 0$ and $H_0 = 0$, $\frac{d}{dH}\frac{V(H)}{H}\Big|_{H=H_0} = -\infty$. Then there must exist some $\bar{H} \in (H_0, \tilde{H})$ such that $\frac{d}{dH}\frac{V(H)}{H}\Big|_{H=\bar{H}} = 0$. Since $\frac{V(H)}{H} > 1$ for all $H \in (H_0, 1)$ by the above argument and since $\tilde{H} \in (H_0, 1)$, we have that $\frac{V(\tilde{H})}{\tilde{H}} > 1$. Since $\lim_{H\to 1}\frac{V(H)}{H} = 1$, it must be true that $\frac{V(H)}{H}$ is decreasing for some $H > \tilde{H}$ and therefore that there exists some $\bar{H}_2 \in (\tilde{H}, 1)$ such that $\frac{d}{dH}\frac{V(H)}{H}\Big|_{H=\bar{H}_2} = 0$. This contradicts the uniqueness of $\bar{H}$ by Lemma 2. Thus, the claim holds.

<u>Case 2: $\alpha > \gamma$, $V_0 = 0$, $H_0 > 0$.</u> Since $V_0 = 0$ and $H_0 > 0$, we have that $c < 0$. Note that $\frac{V(H_0)}{H_0} = \frac{V_0}{H_0} = 0$. By Lemma 1 there exists some unique $H^* \in (H_0, 1)$ such that $\frac{V(H^*)}{H^*} = 1$. Then $\frac{V(H)}{H} < 1$ for $H \in (H_0, H^*)$. We claim that $\frac{V(H)}{H} > 1$ for $H \in (H^*, 1)$. If not, then $V(H) \leq H$ for all $H \in (H_0, 1)$ and by (S21)

$$\frac{d}{dH}\frac{V(H)}{H} = \frac{\alpha H\left(\frac{1}{V}-1\right) - \gamma V\left(\frac{1}{H}-1\right)}{\gamma H(1-H)}$$
$$\geq \frac{\alpha H\left(\frac{1}{H}-1\right) - \gamma H\left(\frac{1}{H}-1\right)}{\gamma H(1-H)} = \frac{\alpha-\gamma}{\gamma H} > 0 \qquad \text{(S24)}$$

since $\alpha > \gamma > 0$. Then since $\frac{V(H^*)}{H^*} = 1$, there must exist some $\tilde{H} \in (H^*, 1)$ such that $\frac{V(\tilde{H})}{\tilde{H}} > 1$, a contradiction to that $V(H) \leq H$ for all $H \in (H_0, 1)$. Thus the claim holds.

Now we examine the sign of $\frac{d}{dH}\frac{V(H)}{H}$. First, we claim that $\frac{d}{dH}\frac{V(H)}{H} > 0$ for all $H \in (H_0, H^*]$. Note that for $H \in (H_0, H^*]$, $H \geq V(H)$. Then the claim holds by (S24).

Next we claim that there exists some $\bar{H} \in (H^*, 1)$ such that $\frac{d}{dH}\frac{V(H)}{H} > 0$ for all $H \in (H_0, \bar{H})$ and $\frac{d}{dH}\frac{V(H)}{H} < 0$ for all $H \in (\bar{H}, 1)$. Indeed, by the above argument, $\frac{d}{dH}\frac{V(H)}{H}\Big|_{H=H^*} > 0$. Recall that $\frac{V(H^*)}{H^*} = 1$. Then there exists some $H_1 \in (H^*, 1)$ such that $\frac{V(H_1)}{H_1} > 1$. Then since $\lim_{H\to 1}\frac{V(H)}{H} = 1$

there must exist some $H_2 \in (H_1, 1)$ such that $\frac{d}{dH}\frac{V(H)}{H}\Big|_{H=H_2} < 0$. Then by the continuity of $\frac{d}{dH}\frac{V(H)}{H}$ there exists some $\bar{H} \in (H^*, H_2) \subset (H^*, 1)$ such that $\frac{d}{dH}\frac{V(H)}{H}\Big|_{H=\bar{H}} = 0$. By Lemma 2 this $\bar{H}$ is unique. Thus the claim holds.

Case 3: $\alpha = \gamma,\ V_0 > 0,\ H_0 = 0.$ Since $V_0 > 0$ and $H_0 = 0$, we have that $c > 0$. Note that $\frac{V(H_0)}{H_0} = \frac{V_0}{H_0} = \infty$. Then since $\alpha = \gamma$, $\frac{V(H)}{H} > 1$ for all $H \in (H_0, 1)$ by Lemma 1.

We claim that $\frac{d}{dH}\frac{V(H)}{H} < 0$ for all $H \in (H_0, 1)$. Since $V(H) > H$ for all $H \in (H_0, 1)$,

$$\alpha H\left(\frac{1}{V} - 1\right) - \gamma V\left(\frac{1}{H} - 1\right) < \alpha H\left(\frac{1}{H} - 1\right) - \gamma H\left(\frac{1}{H} - 1\right) = \alpha - \gamma = 0.$$

then the claim holds by (S21).

Case 4: $\alpha = \gamma,\ V_0 = 0,\ H_0 < 0.$ Since $V_0 = 0$ and $H_0 > 0$, we have that $c < 0$. Note that $\frac{V(H_0)}{H_0} = \frac{V_0}{H_0} = 0$. Then since $\alpha = \gamma$, $\frac{V(H)}{H} < 1$ for all $H \in (H_0, 1)$ by Lemma 1. By an argument similar to that in Case 3, $\frac{d}{dH}\frac{V(H)}{H} > 0$ for all $H \in (H_0, 1)$.

Case 5: $\alpha < \gamma,\ V_0 > 0,\ H_0 = 0.$ Since $V_0 > 0$ and $H_0 = 0$, we have that $c > 0$. Note that $\frac{V(H_0)}{H_0} = \frac{V_0}{H_0} = \infty$. By Lemma 1 there exists some unique $H^* \in (H_0, 1)$ such that $\frac{V(H^*)}{H^*} = 1$. By an argument similar to that in Case 2, we have that $\frac{V(H)}{H} < 1$ for $H \in (H^*, 1)$ and that there exists some $\bar{H} \in (H^*, 1)$ such that $\frac{d}{dH}\frac{V(H)}{H} < 0$ for all $H \in (H_0, \bar{H})$ and $\frac{d}{dH}\frac{V(H)}{H} > 0$ for all $H \in (\bar{H}, 1)$.

Case 6: $\alpha < \gamma,\ V_0 = 0,\ H_0 > 0.$ Since $V_0 = 0$ and $H_0 > 0$, we have that $c < 0$. Note that $\frac{V(H_0)}{H_0} = \frac{V_0}{H_0} = 0$. Then since $\alpha < \gamma$, $\frac{V(H)}{H} < 1$ for all $H \in (H_0, 1)$ by Lemma 1. By an argument similar to that in Case 1, $\frac{d}{dH}\frac{V(H)}{H} \geq 0$ for all $H \in (H_0, 1)$.

We summarize the above cases in Table 3 in Text S3 and illustrate them in Figures S2 and 1.

Table 3 in Text S3. Summary of possible characterizations of $\frac{\mathbf{V(H)}}{\mathbf{H}}$ with all possible cases listed.

	α, γ	**Initial Conditions**	$\frac{V(H)}{H}$	$\frac{d}{dH}\frac{V(H)}{H}$
Case 1	$\alpha > \gamma$	$V_0 > 0,\ H_0 = 0,$ $\lim_{V \to V_0, H \to H_0} \frac{V(H)}{H} = \infty$	$\frac{V(H)}{H} > 1 \forall H \in (H_0, 1)$	$\frac{d}{dH}\frac{V(H)}{H} \leq 0 \,\forall\, H \in (H_0, 1)$
Case 2	$\alpha > \gamma$	$V_0 = 0,\ H_0 > 0,$ $\frac{V_0}{H_0} = 0$	$\exists!\, H^* \in (H_0, 1)$ s.t. $\begin{cases} \frac{V(H)}{H} < 1 \forall H \in (H_0, H^*) \\ \frac{V(H^*)}{H^*} = 1 \\ \frac{V(H)}{H} > 1 \forall H \in (H^*, 1) \end{cases}$	$\exists!\, \bar{H} \in (H^*, 1)$ s.t. $\begin{cases} \frac{d}{dH}\frac{V(H)}{H} > 0 \forall H \in (H_0, \bar{H}) \\ \frac{d}{dH}\frac{V(\bar{H})}{H} = 0 \\ \frac{d}{dH}\frac{V(H)}{H} < 0 \forall H \in (\bar{H}, 1) \end{cases}$
Case 3	$\alpha = \gamma$	$V_0 > 0,\ H_0 = 0,$ $\lim_{V \to V_0, H \to H_0} \frac{V(H)}{H} = \infty$	$\frac{V(H)}{H} > 1 \forall H \in (H_0, 1)$	$\frac{d}{dH}\frac{V(H)}{H} < 0 \,\forall\, H \in (H_0, 1)$
Case 4	$\alpha = \gamma$	$V_0 = 0,\ H_0 > 0,$ $\frac{V_0}{H_0} = 0$	$\frac{V(H)}{H} < 1 \forall H \in (H_0, 1)$	$\frac{d}{dH}\frac{V(H)}{H} > 0 \,\forall\, H \in (H_0, 1)$
Case 5	$\alpha < \gamma$	$V_0 > 0,\ H_0 = 0,$ $\lim_{V \to V_0, H \to H_0} \frac{V(H)}{H} = \infty$	$\exists!\, H^* \in (H_0, 1)$ s.t. $\begin{cases} \frac{V(H)}{H} > 1 \,\forall\, H \in (H_0, H^*) \\ \frac{V(H^*)}{H^*} = 1 \\ \frac{V(H)}{H} < 1 \,\forall\, H \in (H^*, 1) \end{cases}$	$\exists!\, \bar{H} \in (H^*, 1)$ s.t. $\begin{cases} \frac{d}{dH}\frac{V(H)}{H} < 0 \,\forall\, H \in (H_0, \bar{H}) \\ \frac{d}{dH}\frac{V(\bar{H})}{H} = 0 \\ \frac{d}{dH}\frac{V(H)}{H} > 0 \,\forall\, H \in (\bar{H}, 1) \end{cases}$
Case 6	$\alpha < \gamma$	$V_0 = 0,\ H_0 > 0,$ $\frac{V_0}{H_0} = 0$	$\frac{V(H)}{H} < 1 \,\forall\, H \in (H_0, 1)$	$\frac{d}{dH}\frac{V(H)}{H} \geq 0 \,\forall\, H \in (H_0, 1)$

S3.3.1 Linear cost function

Now we assume that our cost function is linear:

$$C(s_V, s_H) = a_V + b_V s_V + a_H + b_H s_H.$$

Then

$$C_{s_V} = b_V \quad \text{and} \quad C_{s_H} = b_H.$$

By the analysis in Section S3.3, it is clear that the optimal sampling design is determined by the relative magnitudes of $\frac{V(H)}{H}$ and the constant $\frac{b_V}{b_H}$. We consider only cases 1 and 2 above. The other cases follow similarly.

Case 1: $\alpha > \gamma$, $V_0 > 0$, $H_0 = 0$. By the above analysis, $\frac{V(H)}{H} > 1$ and $\frac{d}{dH}\frac{V(H)}{H} \leq 0$ for all $H \in (H_0, 1)$. If $\frac{b_V}{b_H} \leq 1$, then

$$\frac{V(H)}{H} > 1 \geq \frac{b_V}{b_H} = \frac{C_{s_V}}{C_{s_H}}$$

for all $H \in (H_0, 1)$, so by the analysis of Section S3.3, we choose to sample only the vector population. If $\frac{b_V}{b_H} > 1$, then since $\lim_{V \to V_0, H \to H_0} \frac{V(H)}{H} = \infty$, there exists some $\hat{H}$ such that

$$\begin{cases} \frac{V(H)}{H} & > \frac{b_V}{b_H} \;\; \forall \;\; H \in (H_0, \hat{H}) \\ \frac{V(\hat{H})}{\hat{H}} & = \frac{b_V}{b_H} \\ \frac{V(H)}{H} & > \frac{b_V}{b_H} \;\; \forall \;\; H \in (\hat{H}, 1) \end{cases}$$

Note that $\hat{H}$ is unique by Lemma 2. Then by the analysis of Section S3.3, at early times in the epidemic (when $H \in (H_0, \hat{H})$), we should sample only the vector population and at late times (when $H \in (\hat{H}, 1)$) we should sample only the host population. Additionally, there exists some intermediate instant (when $H = \hat{H}$) at which we should sample both the vector and host populations.

Case 2: $\alpha > \gamma$, $V_0 = 0$, $H_0 > 0$. By the above analysis, there exists some unique $H^* \in (H_0, 1)$ such that

$$\begin{cases} \frac{V(H)}{H} & < 1 \;\; \forall \;\; H \in (H_0, H^*) \\ \frac{V(H^*)}{H^*} & = 1 \\ \frac{V(H)}{H} & > 1 \;\; \forall \;\; H \in (H^*, 1) \end{cases}$$

and there exists some unique $\bar{H} \in (H^*, 1)$ such that

$$\begin{cases} \frac{d}{dH}\frac{V(H)}{H} & > 0 \;\; \forall \;\; H \in (H_0, \bar{H}) \\ \frac{d}{dH}\frac{V(\bar{H})}{\bar{H}} & = 0 \\ \frac{d}{dH}\frac{V(H)}{H} & < 0 \;\; \forall \;\; H \in (\bar{H}, 1) \end{cases}$$

Then $\frac{V(H)}{H}$ achieves a unique maximum at $\bar{H} \in (H_0, 1)$. If

$$\frac{V(\bar{H})}{\bar{H}} < \frac{b_V}{b_H}$$

then

$$\frac{V(H)}{H} < \frac{b_V}{b_H} = \frac{C_{s_V}}{C_{s_H}}$$

for all $H \in (H_0, 1)$. By the analysis in Section S3.3, we choose to sample only the host population. If

$$\frac{V(\bar{H})}{\bar{H}} > \frac{b_V}{b_H}$$

then there exist some $H_1, H_2 \in (H_0, 1)$, $H_1 < H_2$ such that

$$\begin{cases} \frac{V(H)}{H} & < \frac{b_V}{b_H} \ \forall \ H \in (H_0, H_1) \\ \frac{V(H_1)}{H_1} & = \frac{b_V}{b_H} \\ \frac{V(H)}{H} & > \frac{b_V}{b_H} \ \forall \ H \in (H_1, H_2) \\ \frac{V(H_2)}{H_2} & = \frac{b_V}{b_H} \\ \frac{V(H)}{H} & < \frac{b_V}{b_H} \ \forall \ H \in (H_2, 1) \end{cases}$$

Then at early stages of the epidemic (while $H \in (H_0, H_1)$), we should sample only the host population, at intermediate times (while $H \in (H_1, H_2)$) we sample only the vector population, and at late times in the epidemic (while $H \in (H_2, 1)$) we return to sampling only host population. As in Case 1, if $H = H_1$ or $H = H_2$, then we should sample both the vector and host populations.

As in the main text, we find that there is a critical time at which we should switch our sampling scheme. We can solve for this critical time numerically.
