# Supplementary material for: Optimal Sampling Strategies for Detecting Zoonotic Disease Epidemics
Source: PLoS Comput Biol. 2014 Jun 26;10(6):e1003668. doi: 10.1371/journal.pcbi.1003668 (PMC4072525; doi:10.1371/journal.pcbi.1003668)
Supplement: Text S4 — Critical time for alternative initial conditions. Derivation of critical times for when the initial infected individual is in the vector population. (PDF) [file pcbi.1003668.s006.pdf]

S4 Critical times for alternative initial conditions

S4.1 SI host- SI vector model

In the case where the initial conditions are of the form ($I_V(0) > 0$, $I_H(0) = 0$), solutions to the SI host- SI vector model (2) are:

$$I_V(t) = I_V(0)\cosh(\lambda t) \tag{S25a}$$

$$I_H(t) = I_V(0)\frac{\beta_{H,V}N_H}{\lambda N_V}\sinh(\lambda t), \tag{S25b}$$

where as before $\lambda = \sqrt{\beta_{V,H}\beta_{H,V}}$. The critical time for this system can be solved by determining when the economic efficiencies become equal by plugging in solutions from (S25) into the economic efficiency conditions given in (17) and solving for $\tau_{V\to H}$.

$$\begin{aligned}
\eta_H(\tau_{V\to H}) &= \eta_V(\tau_{V\to H}) \\
\frac{I_H(\tau_{V\to H})}{N_H b_H} &= \frac{I_V(\tau_{V\to H})}{N_V b_V} \\
\frac{I_V(0)\frac{\beta_{H,V}N_H}{\lambda N_V}\sinh(\lambda t)}{N_H b_H} &= \frac{I_V(0)\cosh(\lambda\tau_{V\to H})}{N_V b_V} \\
\frac{\sinh(\lambda\tau_{V\to H})}{\cosh(\lambda\tau_{V\to H})} &= \frac{\lambda b_H}{\beta_{H,V}b_V} \\
\tanh(\lambda\tau_{V\to H}) &= \frac{\lambda b_H}{\beta_{H,V}b_V} \\
\tau_{V\to H} &= \frac{1}{\lambda}\text{atanh}\left(\frac{\lambda b_H}{\beta_{H,V}b_V}\right).
\end{aligned}$$

S4.2 SIR host- SI vector model

In the case where the host population follows the linearized SIR dynamics given in (5) with initial conditions ($I_V(0) > 0$, $I_H(0) = 0$, $R_H(0) = 0$) solutions are:

$$I_V(t) = I_V(0)e^{-\gamma t/2}\left(\cosh(\mu t/2) + \frac{\gamma}{\mu}\sinh(\mu t/2)\right) \tag{S26a}$$

$$I_H(t) = I_V(0)\frac{2\beta_{H,V}N_H}{N_V\mu}e^{-\gamma t/2}\sinh(\mu t/2) \tag{S26b}$$

$$R_h(t) = I_V(0)\frac{\gamma N_H}{\beta_{V,H}N_V}\left(e^{-\gamma t/2}\cosh(\mu t/2) + \frac{\gamma e^{-\gamma t/2}}{\mu}\sinh(\mu t/2) - 1\right) \tag{S26c}$$

As before $\mu = \sqrt{\gamma^2 + 4\beta_{H,V}\beta_{V,H}}$. We consider the three critical times; $\tau_{V\to H}$, $\tau_{H\to R}$, and $\tau_{V\to R}$. The time at which the optimal sampling strategy switches from sampling only the vector population to sampling and testing only infected hosts is given by $\tau_{V\to H}$. The time at which the optimal sampling strategy switches from sampling and testing infected hosts to sampling and testing recovered hosts is given by $\tau_{H\to R}$. Lastly, $\tau_{V\to R}$ is the time at which the optimal sampling strategy switches from sampling only the vector population to sampling and testing recovered hosts.

For the first case, we consider testing between infected vectors and infected hosts, similar to the SI model in the previous section. The critical time $\tau_{V\to H}$ is given as before by equating the economic efficiencies at the critical time, $\eta_H(\tau_{V\to H}) = \eta_V(\tau_{H\to R})$. Plugging in $I_V(\tau_{V\to H})$ and $I_H(\tau_{V\to H})$ from (S26) and solving for $\tau_{V\to H}$ gives

$$\tau_{V\to H} = \frac{2}{\mu}\text{atanh}\left(\frac{\mu b_H}{2\beta_{H,V}b_V - \gamma b_H}\right). \tag{S27}$$

The second critical time, a switch in sampling from infected hosts to recovered hosts, is given when $\eta_H(\tau_{H\to R}) = \eta_R(\tau_{H\to R})$. Plugging in solutions from (S26) gives,

$$\frac{2b_R\beta_{HV}\beta_{VH}}{b_H\gamma\mu} - \frac{\gamma}{\mu} = \coth\left(\frac{\mu\tau_{H\to R}}{2}\right) - e^{\gamma\tau_{H\to R}/2}\text{csch}\left(\frac{\mu\tau_{H\to R}}{2}\right). \tag{S28}$$

For early times we can do a first order Taylor series expansion around $\tau_{H\to R} = 0$, where

$$\coth\left(\frac{\mu\tau_{H\to R}}{2}\right) \approx \frac{2}{\mu\tau_{H\to R}} + \frac{\mu\tau_{H\to R}}{6}$$

and

$$e^{\gamma\tau_{H\to R}/2}\text{csch}\left(\frac{\mu\tau_{H\to R}}{2}\right) \approx \left(1 + \frac{\gamma\tau_{H\to R}}{2}\right)\left(\frac{2}{\mu\tau_{H\to R}} - \frac{\mu\tau_{H\to R}}{12}\right).$$

Plugging these approximations into (S28) gives

$$\begin{aligned}
\frac{2b_R\beta_{HV}\beta_{VH}}{b_H\gamma\mu} - \frac{\gamma}{\mu} &\approx \frac{2}{\mu\tau_{H\to R}} + \frac{\mu\tau_{H\to R}}{6} - \left(1 + \frac{\gamma\tau_{H\to R}}{2}\right)\left(\frac{2}{\mu\tau_{H\to R}} - \frac{\mu\tau_{H\to R}}{12}\right)\\
\frac{2b_R\beta_{HV}\beta_{VH}}{b_H\gamma\mu} - \frac{\gamma}{\mu} &\approx \frac{\mu\tau_{H\to R}}{4} - \frac{\gamma}{\mu} + \frac{\gamma\mu\tau_{H\to R}^2}{24}\\
\frac{2b_R\beta_{HV}\beta_{VH}}{b_H\gamma\mu} &\approx \frac{\mu\tau_{H\to R}}{4} + \frac{\gamma\mu\tau_{H\to R}^2}{24}.
\end{aligned} \tag{S29}$$

This quadratic for $\tau_{H\to R}$ can be solved to get the critical time;

$$\begin{aligned}
\tau_{H\to R} &\approx \frac{-\frac{\mu}{4} \pm \sqrt{\frac{\mu^2}{4^2} + 4\frac{\gamma\mu}{24}\frac{2b_R\beta_{HV}\beta_{VH}}{b_H\gamma\mu}}}{\frac{\gamma\mu}{12}}\\
\tau_{H\to R} &\approx -\frac{3}{\gamma} \pm \frac{12}{\gamma\mu}\sqrt{\frac{\mu^2}{4^2} + 4\frac{\gamma\mu}{24}\frac{2b_R\beta_{HV}\beta_{VH}}{b_H\gamma\mu}}\\
\tau_{H\to R} &\approx -\frac{3}{\gamma} \pm \frac{3}{\gamma}\sqrt{1 + \frac{16}{3}\frac{b_R\beta_{V,H}\beta_{H,V}}{b_H\mu^2}},
\end{aligned}$$

where we are interested in the positive root.

The third and final case is the switch from infected vectors to recovered hosts. In this case we set equal the economic efficiencies for vectors and recovered hosts, $\eta_V(\tau_{V\to R}) = \eta_R(\tau_{V\to R})$. This gives the following expression,

$$e^{-\gamma\tau_{V\to R}/2}\left(\cosh(\mu\tau_{V\to R}/2) + \frac{\gamma}{\mu}\sinh(\mu\tau_{V\to R}/2)\right) = \frac{b_V\gamma}{b_V\gamma - b_R\beta_{V,H}}. \tag{S30}$$

Again, this equation is not analytically solvable for $\tau_{V\to R}$ but can be further approximated. A first order Taylor expansion around $\tau_{V\to R} = 0$ gives the following approximations;

$$\begin{aligned}
e^{-\gamma\tau_{V\to R}/2} &\approx 1 - \gamma\tau_{V\to R}/2\\
\cosh(\mu\tau_{V\to R}/2) &\approx 1\\
\sinh(\mu\tau_{V\to R}/2) &\approx \mu\tau_{V\to R}/2.
\end{aligned}$$

Plugging these approximations into (S30) gives

$$\begin{aligned}\left(1-\frac{\gamma\tau_{V\to R}}{2}\right)\left(1+\frac{\gamma}{\mu}\frac{\mu\tau_{V\to R}}{2}\right) &\approx \frac{b_V\gamma}{b_V\gamma-b_R\beta_{V,H}}\\ 1-\frac{\gamma^2\tau_{V\to R}^2}{4} &\approx \frac{b_V\gamma}{b_V\gamma-b_R\beta_{V,H}}\\ \tau_{V\to R} &\approx \frac{2}{\gamma}\sqrt{\frac{b_R\beta_{V,H}}{b_R\beta_{V,H}-b_V\gamma}}.\end{aligned} \tag{S31}$$
